# Gamma Radiation- and Ultraviolet-Induced Polymerization of Bis(amino acid)fumaramide Gel Assemblies

**DOI:** 10.3390/polym14010214

**Published:** 2022-01-05

**Authors:** Tomislav Gregorić, Janja Makarević, Zoran Štefanić, Mladen Žinić, Leo Frkanec

**Affiliations:** 1 Laboratory of Supramolecular Chemistry, Division of Organic Chemistry and Biochemistry, Ruđer Bošković Institute, 10000 Zagreb, Croatia; tgregor@irb.hr (T.G.); Janja.Makarevic@irb.hr (J.M.); zinic@hazu.hr (M.Ž.); 2 Laboratory for Chemical and Biological Crystallography, Division of Physical Chemistry, Ruđer Bošković Institute, 10000 Zagreb, Croatia; Zoran.Stefanic@irb.hr; 3 Croatian Academy of Sciences and Arts, Trg Nikole Šubića Zrinskog 11, 10000 Zagreb, Croatia

**Keywords:** radiation-induced polymerization, photoinduced polymerization, self-assembly, low-molecular-weight gelators (LMWG), topochemical polymerizations, polymer-based gelators

## Abstract

Controlling the polymerization of supramolecular self-assembly through external stimuli holds great potential for the development of responsive soft materials and manipulation at the nanoscale. Vinyl esters of bis(leu or val)fumaramide (**1a** and **2a**) have been found to be gelators of various organic solvents and were applied in this investigation of the influence of organogelators’ self-assembly on solid-state polymerization induced by gamma and ultraviolet irradiation. Here, we report our investigation into the influences of self-assemblies of bis(amino acid vinyl ester)fumaramides on gamma-ray- and ultraviolet-induced polymerization. The gelator molecules self-assembled by non-covalent interactions, mainly through hydrogen bonds between the amide group (CONH) and the carboxyl group (COO), thus forming a gel network. NMR and FTIR spectroscopy were used to investigate and characterize supramolecular gels. TEM and SEM microscopy were used to investigate the morphology of gels and polymers. Morphology studies showed that the gels contained a filamentous structure of nanometer dimensions that was exhaustive in a three-dimensional network. The prepared derivatives contained reactive alkyl groups suitable for carrying out the polymerization reaction initiated by gamma or ultraviolet radiation in the supramolecular aggregates of selected gels. It was found that the polymerization reaction occurred only in the network of the gel and was dependent on the structure of aggregates or the proximity and orientation of double bonds in the gel network. Polymers were formed by the gels exposure to gamma and ultraviolet radiation in toluene, and water/DMF gels with transcripts of their gel structure into polymers. The polymeric material was able to immobilize various solvents by swelling. Furthermore, methyl esters of bis(leu and val)fumaramide (**1b** and **2b**) were synthesized; these compounds showed no gelling properties, and the crystal structure of the valine derivative **2b** was determined.

## 1. Introduction

**Figure 1:**
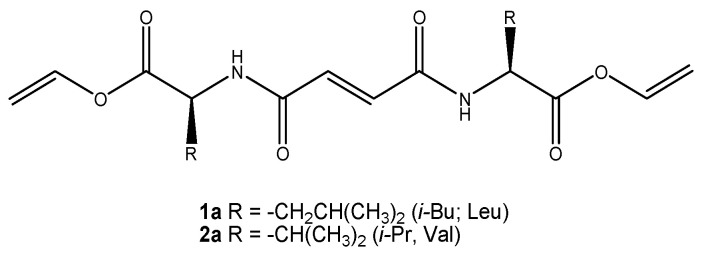
Chemical structure of bis(amino acid)formamide vinyl esters organogelators.

Supramolecular gels are a class of self-assembled soft nanomaterials whose design has been a great challenge over the last few decades. Efforts have been made to improve their gelling properties for specific applications, and to develop new materials [1,2,3,4,5,6]. It has been shown that even short peptides, such as dipeptides, tripeptides and tetrapeptides, by themselves or incorporated into more complex structures, are capable of self-assembling into fibers or fibrils [7,8,9,10,11]. Small molecule gelators represent remarkable examples of molecular self-assembly. The aggregation of gelator molecules into fibrous networks is driven by multiple weak non-covalent interactions, such as hydrogen bonds, π–π stacking, van der Waals forces, electrostatic, and charge-transfer interactions. The supramolecular gel is a reversible system, which distinguishes supramolecular gels from polymer gels. The latter have three-dimensional structures created by crosslinked covalent bonds. In gels, the fibers are heavily entangled into three-dimensional networks that immobilize solvents and prevent fluidity in the system. Such compounds make networks of fibers that can immobilize up to 10^5^ liquid molecules per gelator and increase the viscosity of organic media by factors of up to 10^10^, with the potential to respond to a variety of stimuli [12,13,14,15,16,17,18,19,20]. The morphology of gel-networks can be very diverse (e.g., fibers, tubes, ribbons, and vesicles) as a result of the specific relationship of the gelator structure and the properties of the solvent. Due to the well-defined nano/micro gel-network structure and a diversity of morphologies, organogelators can be a source for the successful preparation of new soft functional nanomaterials [21,22,23,24,25,26,27,28,29,30]. Morphology is also one of the major factors that define the hardness and elasticity of supramolecular gels as semisolid materials. Supramolecular gels are softer and less stable than chemical (polymer) gels due to the nature of noncovalent interactions, but they can be stabilized by the covalent crosslinking of previously formed supramolecular aggregates. In this process, the first step is the self-assembly of organogelator molecules through noncovalent interactions into three-dimensional elongated aggregates that entangle, leading to a network that is capable of immobilizing the present solvent (gelation). The final step is the stabilization of the supramolecular organization by the covalent crosslinking of appropriate functional groups of self-assembled monomers stimulated by various means. These functional groups must be close enough such that polymerization does not cause a disruption of the existing self-organization [31,32,33,34]. Covalent linking usually significantly improves the thermal and mechanical stability of supramolecular gels. In a select few cases, polymerization had no significant influences on the gel properties [35]. Polymerizations of ethylene [36,37,38,39] or acetylene [40,41,42,43,44,45] organogelators, mostly induced by UV irradiation, have been described. Due to photoactive units, fumaramides are very interesting and widely investigated compounds in supramolecular chemistry. For example, fumaramide derivatives (see Figure 1) containing photocontrollable rotaxane were studied by computational and experimental means [46]. We previously investigated the cis–trans photoisomerization of stilbene derivatives [47,48] and maleic acid amide to the fumaramide isomerization, which resulted in a morphological transition at the supramolecular level comprising the transformation of the maleic acid amide microspheres into the fumaramide hydrogel [49]. The most studied reactions were isomerization [50,51,52,53,54,55] and cycloadditions of fumarate, because the 1,2-disubstituted ethylene moiety could be polymerized in the solid-state and in solution but at lower polymerization rates than acetylene, dienes, or vinyl monomers. Fumaric acid derivatives could be transformed by photo [56,57,58], microwave [59], thermal [60], and γ-ray induced polymerizations [61,62], but the polymerization of self-assembled fumaramide molecules in the gel state has not yet been described. Of the above examples, it was deemed necessary to highlight the pioneering research reported by the Feringa group on photopolymerizable organogelators, based on (1*R*,2*R*)-*trans*-1,2-bis(ureido)cyclohexane: i.e., the formation of highly stable organogels with a transcription of the gel network into the polymer [40], the gel stabilization by the polymerization of low-molecular-mass gelators with diyne functional groups, [43] and the stabilization of organogels by click chemistry polymerization in a supramolecular environment [63]. The main aim of this work was the study of the gamma-radiation- and ultraviolet-induced polymerization reaction of bis(amino acid)fumaramide derivatives in gel assemblies with a transcription of the three-dimensional gel network to the polymer.

## 2. Materials and Methods

### 2.1. General Methods

**Scheme 1:**
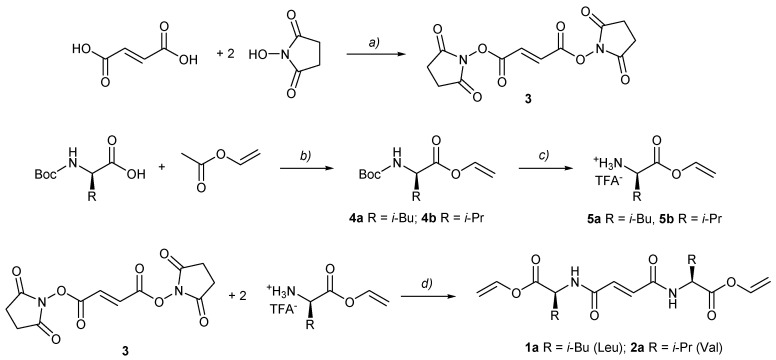
Synthesis of gelators **1a** and **2a** (bis(amino acid)formamide vinyl esters) from *N*-Boc-protected amino acids. (**a**) TFAA, Py, DMF, r.t.; (**b**) Pd(OAc)_2_, K_2_CO_3_, *p*-Benzoquinone, r.t.; (**c**) TFA/CH_2_Cl_2,_ 0 °C; (**d**) Et_3_N, DMF, r.t.

Melting points were determined on Kofler stage and left uncorrected. ^1^H and ^13^C NMR spectra were recorded with Bruker Avance DPX 300 and 600 spectrometers (300/75 Hz and 600/150 MHz) Bruker BioSpin GmbH, Ettlingen, Germany. TMS was used as internal standard. TLC was performed on SilicaGel Merck 60 F254 silica plates, and column chromatography was carried out using 230 ± 240 mesh Merck 60 silica gel (Merck KGaA, Darmstadt, Germany). FTIR spectra were recorded on Bruker Bomen MB 102 spectrometers (Bruker Optics GmbH & Co. KG, Bremen, Germany). Optical rotation was measured on an AA-10 polarimeter (Optical Activity Limited, Ramsey, Cambridgeshire, UK) at a wavelength of 589.3 nm. UV-vis spectra were recorded on a Varian Cary 100 Bio spectrophotometer (Agilent Technologies, Inc., Santa Clara, CA, USA). The gel morphology was determined by transmission electron microscopy (TEM, Zeiss EM 10A, Carl Zeiss AG, Oberkochen, Germany) acceleration voltage 60 kV). The morphology of the polymer was determined by scanning electron microscopy (SEM) on a high-resolution scanning electron microscope (JSM 7000F, manufactured by JEOL Ltd., Tokyo, Japan). The high-resolution mass spectra of the synthesized compounds were recorded with a MALDI-TOF/TOF tandem device (matrix-assisted laser radiation desorption ionization-mass time analyzer) 4800 Plus MALDI TOF/TOF (Applied Biosystems Inc., Foster City, CA, USA). All chemicals were of the best grade commercially available and were used without purification. Solvents were purified according to standard procedures; dry solvents were obtained according to literature methods and stored over molecular sieves [64]. Gel-to-sol temperature (*T*_gel_), or thermal gel-to-sol phase transition behavior of the prepared gel, was observed by a typical tube inversion experiment. In the tube inversion method, the vial was kept in a temperature-controlled water bath with an increasing rate of 1 °C min^−1^. The flow of gel was observed by tilting the vial, and the starting temperature of the gel mass flow was taken as *T*_gel_(K) [65,66]. Gamma radiation of the radionuclide cobalt-60 (60Co) source was used as a polymerization initiator. The dose value was 200 kGy, and the dose rate was 14 kGy h^−1^ or 25 kGy h^−1^. Ultraviolet radiation was carried out with a high-pressure Hg lamp of 100 W with the benzophenone (5%) as photo-initiator. The reaction was carried out in a quartz tube in solvents degassed by ultrasound and bubbled with argon. Compound **3** [67] and compounds **4a** and **4b** [68], used in the synthesis of **1a** and **2a** (Scheme 1) and compounds **1b** [69] and **2b** [70] were prepared according to the procedures described in the literature.

### 2.2. Synthesis, General Procedures for the Preparation of Fumaryl Diesters ***1a*** and ***2a***

1. Bis(2,5-dioxopyrolidne-1-yl)-fumarate (**3**).

Prepared according to [67]: fumaric acid (116.1 mg, 1 mmol) and N-hydroxysuccinimide (575.5 mg, 5 mmol) were dissolved in DMF (5 mL) and dry pyridine (1 mL) in an argon atmosphere. The reaction mixture was then placed in an ice bath and trifluoroacetic anhydride (0.84 mL, 6 mmol) was added dropwise. Then, the reaction was allowed to stir overnight at room temperature. The reaction mixture was then concentrated in vacuo, and EtOAc was added to precipitate the product. White powder was obtained (310.2 mg, 49% yield). NMR spectra were in accordance with the literature data [68].

2. Boc-protected amino acid vinyl ester; Vinyl *N*-(tert-butoxcarbonyl) L-leucinate (**4a**) and Vinyl N-(tert-butoxcarbonyl) L-valinate (**4b**).

The fumaryl diesters were prepared by the Pd-catalyzed transvinylization of bis(leu or val)fumaramide with vinyl acetate accordingly to [71]. Boc-protected amino acid Boc-L-leu (231.3 mg, 1.0 mmol) or Boc-L-val (217.3 mg, 1.0 mmol) was dissolved in 4.4 mL (47.2 mmol) vinyl acetate. KOH (5.6 mg, 0.1 mmol), Pd(OAc)_2_ (2.2 mg, 0.01 mmol), and *p*-benzoquinone (2.1 mg, 0.02 mmol) were added sequentially to this vinyl ester mixture at 22 °C. After 36 h of stirring, NaBH_4_ (2.3 mg, 0.06 mmol) was added, and the reaction mixture was stirred for half an hour. The reaction mixture was then filtered through a pad of celite that was washed with EtOAc. Filtrate was concentrated by rotary evaporation; remaining oil was dissolved in EtOAc, washed with saturated NaHCO_3_(aq) and saturated brine and dried over MgSO_4_. The crude product was isolated by rotary evaporation and purified by column chromatography (hexanes:EtOAc, 75:25 *v/v*) followed by vacuum distillation to yield a yellow-to-colorless oil (60% yield). NMR spectra of the resulting products (**4a** and **4b**) were in accordance with the literature data [68].

3. Deprotection of Boc group, amino acid vinyl ester (general procedure); L-leucine vinyl ester trifluoroacetic acid (**5a**) and L-valine vinyl ester trifluoroacetic acid (**5b**).

The previously synthesized vinyl esters of amino acids leu **4a** (241.9 mg, 1 mmol) and val **4b** (241.9, 1 mmol) were dissolved in 5 mL dry dichloromethane (CH_2_Cl_2_) and placed in an ice bath. Trifluoroacetic acid (TFA, 1 mL) was added and stirred for 1 h at 0 °C and for another 3 h at room temperature. The reaction was followed by TLC chromatography. After completion of deprotection, the solvents were evaporated in vacuo. Then, the amino acid derivatives **5a** and **5b** were obtained in quantitative yield and used without further purification.

### 2.3. General Procedure for Synthesis Vinyl Ester Bis(leu and val)fumaramides (***1a*** and ***2a***)

We added *N*-hydroxysuccinimide ester **3** (310 mg, 1.0 mmol) to a cooled (−5 °C) solution of amino acid vinyl ester trifluoroacetate (2.1 mmol) and TEA (0.573 mL, 4.1 mmol) in 10 mL dry CH_2_Cl_2_ under nitrogen. The mixture was stirred at −5 °C for 30 min and then overnight at room temperature. The reaction mixture was washed successively with 5% HCl, 5% NaHCO_3_, and water. The organic layer was dried (Na_2_SO_4_) and the solvent evaporated under reduced pressure. The product was purified by chromatography on preparative TLC plates, CH_2_Cl_2_:EtOH (25:1 *v/v*).

4. *N*,*N’*-bis((2S)-1-vinyloxy-4-methyl-1-oxopentane-2-yl)fumaramide (**1a**).

Following the general procedure, the title compound was obtained, starting from (569.6 mg, 2.1 mmol) leucine vinyl ester trifluoroacetate (**4a**).

White powder 221 mg (56%, yield), MP=177–179 °C (CH_3_CN), [α]_D_ = −54,5 (γ = 1 g/mL, MeOH);

^1^H NMR (600 MHz, DMSO-d_6_, 20 °C): δ = 8.95 (2H, d, *J* = 6 Hz NH), 7.21 (2 H, dd, *J* = 6.2, *J* = 13.9, OC**H**=CH_2_), 6.92 (2H, s, HC=CH), 4.96 (2H, dd, *J* = 1.9, *J* = 13.9, OCH=C**H_A_**H_B_), 4.73 (2H, dd, *J* = 1.9, *J* = 6.2, OCH=CH_A_**H_B_**), 4.43–4.39 (2 H, m, CH_α_), 1.67–1.56 (6 H, m, CH_2,__β_ and CH _γ_), 0.92 (6H, dd, *J* = 6.2, CH_3,δ_), 0.87 (6H, dd, *J* = 6.2, CH_3,δ_) ppm. ^13^C NMR (75 MHz, DMSO-d_6_, 20 °C): δ=170.1 (COO), 164.3 (CON), 141.7 (O**C**H=CH_2_) 132.9 (HC=CH), 99.6 (OCH=**C**H_2_), 51.0 (CH_α_), 39.6 (CH_2,__β_), 24.8 (CH_γ_), 23.1, 21.6 (CH_3,δ_) ppm.; FTIR (KBr) v_max_/cm^−1^ = 3320 (NH), 1755 (OC=O), 1630 (HNC=O, amide I), 1538 (HNC=O, amide II). HRMS: *m*/*z* [M+Na)]^+^: C_20_H_30_N_2_O_6_, calculate: 417.2002, found: 417.2006.

5. *N*,*N*’-bis((2*S*)-1-vinyloxy-3-methyl-1-oxobutane-2-yl)fumaramide (**2a**).

Following the general procedure, the title compound was obtained, starting from (540.14 mg, 2.1 mmol) valine vinyl ester trifluoroacetate (**4b**).

White powder 205 mg (55.9%, yield). MP = 175–176 °C (CH_3_CN), [α]_D_ = −68.5 (γ = 1 g/mL, MeOH);

^1^H NMR (300 MHz, CDCl_3_, 20 °C): δ =7.24 (2 H, dd, *J* = 6.2 Hz, *J* = 13.9 Hz, OC**H**=CH_2_), 7.1 (2H, s, HC=CH), 6.91 (2 H, d, *J* = 9 Hz, NH), 4.95 (2H, dd, *J* = 1.7, *J* = 13.9, OCH=C**H_A_**H_B_), 4.79 (2 H, dd, *J* = 5.0 Hz, *J* = 9.0 Hz, CH_α_), 4.65 (2H, dd, *J* = 1.8 Hz, *J* = 6.2 Hz, OCH=CH_A_**H_B_**), 2.32–2.25 (2 H, m, CH_β_), 1.00 (6H, 2d, *J* = 6.9 Hz, CH_3,δ_); 0.97 (6H, 2d, *J* = 6.9 Hz, CH_3,δ_) ppm.; ^13^C NMR (75.5 MHz, CDCl_3_, 20 °C): δ = 168.9 (COO), 164.8 (CON), 140.8 (O**C**H=CH_2_) 133.3 (HC=CH), 99.0 (OCH=**C**H_2_), 57.1 (CH_α_), 31.4 (CH_2,__β_), 18.9 (CH_3,γ_), 17.7 (CH_3,γ_) ppm.; FTIR (KBr) v_max_/cm^−1^ = 3267 (NH), 1750 (OC=O), 1637 (HNC=O, amide I), 1553 (HNC=O, amide II); HRMS: *m*/*z* [M+H]^+^: C_18_H_26_N_2_O_6_, calculate: 367.1869, found: 367.1870.

### 2.4. General Procedure for Synthesis of Methyl Ester Bis(leu and val)fumaramides (***1b*** and ***2b***)

In a flask-appropriate amino acid, (L-leu or L-val) (2 mmol) was dissolved in dry methanol (25 mL) and cooled to −10 °C. Then, over 30 min, we added, dropwise, SOCl_2_ (0.5 mL, 7 mmol). The reaction was stirred overnight and then another 3 h at 40 °C. The reaction mixture was concentrated and product was precipitated with petroleum ether as white powder. To a cooled (−5 °C) solution of previously synthesized compounds (2 mmol methyl ester hydrochloride L-leu or L-val) and TEA (0.44 mL, 3.1 mmol) in dry CH_2_Cl_2_ (6 mL), a solution of fumaryl chloride (0.11 mL, 1.0 mmol) in CH_2_Cl_2_ (5 mL) was added under nitrogen. The mixture was stirred at −5 °C for 30 min and then overnight at room temperature. The reaction mixture was washed successively with 5% HCl, 5% NaHCO_3_ and water. The organic layer was dried (Na_2_SO_4_) and the solvent evaporated under reduced pressure. The product was purified by preparative thin layer chromatography (PTLC) CH_2_Cl_2_:MeOH (10:1 v/v).

6*. N,N’*-bis((2*S*)-1-methoxy-3-methyl-1-oxobutane-2-yl)fumaramide (**1b**).

Compound **1b** was synthesized according to general procedure. White powder 123.9 mg was obtained in a yield of 45.7%. The NMR spectra of the obtained product corresponded to the data from the literature [69].

Following the general procedure, the title compound was obtained, starting from L-leucine (264 mg, 2 mmol).

^1^H NMR (300 MHz, DMSO-d_6_): δ = 8.80 (2H, d, NH, *J* = 7.7 Hz), 6.90 (2H, s, HC=CH), 4.41–4.34 (2H, m, CHα), 3.32 (6H, s, OCH_3_) 1.63–1.52 (2H, m, CH_β_ and CH_2_), 0.87 (12H, dd, CH3, *J* = 6.2 Hz, *J* = 14.5 Hz) ppm; ^13^C NMR (75 MHz, DMSO-d_6_): δ = 172.6 (C=O), 163.7 (NHC=O), 132.5 (HC=CH), 51.9 (CHα), 50.5 (OCH_3_), 39.8 (CH_2_), 29.6 (CH_β_), 22.6 (CH_3_), 21.1 (CH_3_) ppm.; FTIR (KBr) v_max_/cm^−1^ = 3300 (NH), 1751 (OC=O), 1732 (OC=O), 1633 (HNC=O, amide I), 1534 (HNC=O, amide II).

7*. N,N’*-bis((2S)-1-methoxy-4-methyl-1-oxopentane-2-yl)fumaramide (**2b**) 

Compound **2b** was synthesized according to general procedure starting from L-valine (234 mg, 2 mmol). White powder (125 mg) was obtained with a yield of 53.5%. The spectral data were consistent with those reported in the literature. [70]. A single crystal **1b** for X-ray diffraction analysis was obtained by recrystallization and slow evaporation from methanol (MeOH). Crystallographic data of 2**b** were deposited in the Cambridge Crystallographic Data Centre under accession number CCDC: 2124266.

## 3. Results and Discussion

### 3.1. Molecular Design and Synthesis of Fumaramide Gelators

The bis-amino acid (val and leu)fumaramide derivatives (**1a, 2a**) were designed with two different alkene groups (–OC=CO-. –O-CH=CH_2_). Both of these could be polymerized, while the core of the molecule bearing two amide groups enabled the formation of strong intermolecular hydrogen bonds. Designed fumaramide derivatives possess relatively rigid molecular architectures and preorganized geometries for the hydrogen-bond-governed unidirectional self-assembly into complex nanofibrillar structures by gelling various solvents. Fumaramides are centrosymmetric and self-complementary molecules. In the crystal lattice of bis(leu-OMe)fumaramide, a typical self-organization motif through intermolecular hydrogen bonding between central fumaramide units (-NH-CO=CO-NH-) was observed [69]. Similar organizational motifs were also found in the self-assemblies of oxalamide gelators that were previously studied in detail [49]. We assumed that the self-assembly of vinyl ester valine and leucine fumaramide organogelators (**1a** or **2a**, Figure 1) could be polymerized under the significant influence of both the gelator molecule packing in the assemblies and the morphology of the gel network.

The self-assembly motifs of **1a** and **2a** with an appropriate orientation of vinyl ester units may have enabled the polymerization through a central fumaryl moiety and vinyl ester group. The solvent properties and lipophilic groups at the asymmetric centers can have a significant influence on the organogelator self-assembly. The detailed synthetic routes for the preparation of chiral bis(amino acid)formamide vinyl esters (**1a**, **2a**) are summarized in Scheme 1. All of the novel structures were unambiguously confirmed by ^1^H and ^13^C NMR, FTIR and high-resolution ESI-MS. The synthesis and analytical characterization of the prepared compounds are collected in the Experiment section and Supporting Information.

The vinyl esters of amino acids (**4a, 4b**) were prepared by the Pd-catalyzed transvinylation directly from *N*-protected amino acid derivatives. Gelators, fumaric derivatives (**1a, 2a**), were prepared from fumaryl disuccimide (**3**) and valine and leucine vinyl esters, respectively, with moderate yields. The overall reaction yields for gelators **1a** and **2a** were around 50%. We noted that, prior to purification, the yield was around 80%; however, due to isolation, yields were down by about 30%. After purification, both vinyl esters were stable for a long time at 2–8 °C.

### 3.2. Gelation Properties

The investigated fumaramides **1a** and **2a** were low-to-moderately efficient gelators. Leucine derivative **1a** could immobilize solvents of different polarity, but the gelation was mostly induced by ultrasound. Of the investigated solvents, **2a** gelled only toluene and the DMSO/H_2_O solvent mixture. The results of the gelation experiments are given in the (Table 1).

### 3.3. FTIR and ^1^H NMR Investigations of ***1a*** and ***2a*** Self-Assemblies, and Molecular Modeling

To identify the supramolecular interactions that stabilize gel assemblies, the selected gels were studied by ^1^H NMR, and FTIR spectroscopy. Valuable information on the self-assembly of gelator molecules in the pre-gelation and gel states were obtained by the analysis of their concentration and temperature-dependent ^1^H NMR and FTIR spectra. It was previously reported that the planar and self-complementary fumarylalamide unit persistently formed intermolecular hydrogen bonds and represented the major organizational element in the gel assemblies of bis(amino acid)fumaramides, while also exerting a major influence on their organization in the solid state [50,70]. The spectroscopic investigation of the self-assemblies of **1a** and **2a** in toluene suggested that hydrogen bonding was the most important process, dictating the organization of both organogelators. In the FTIR spectra of **1a**/toluene gel, NH bands appeared at 3288 cm^−1^ and two NH bands appeared at 3266 and 3291 cm^−1^ in **2a**/toluene gels, corresponding to the hydrogen bonded NHs as well as the amide I bands at 1638 (**1a**) and 1635 cm^−1^ (**2a**).

**Figure 2:**
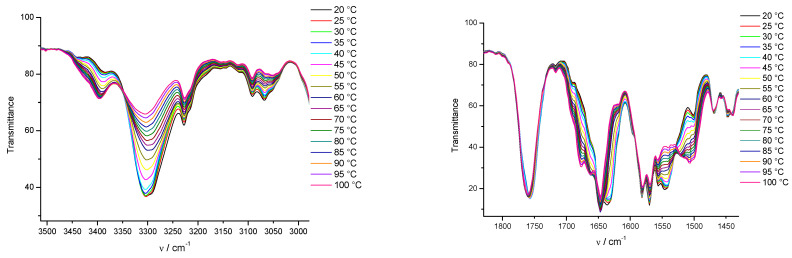

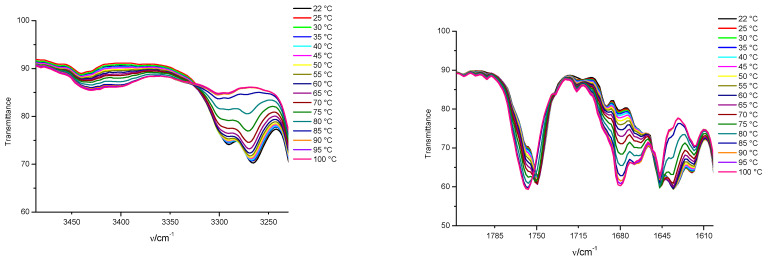
**Upper** plots: temperature-dependent FTIR spectra of **1a**/toluene-d_8_ (c = 0.23 M) and **Lower** plots: temperature-dependent FTIR spectra of **2a**/toluene-d_8_ (c = 4.2 × 10^−2^ M) gels in the temperature range of 20 (22)–100 °C.

By the gel-to-sol transition, the bands were shifted to 3396, 1678 cm^−1^ (**1a**) and 3402, 1679 cm^−1^ (**2a**), respectively (Figure 2). The carboxyl starching band of **1a** organogelators appeared at 1760 cm^−1^ and was not shifted by the increasing temperature. These results indicate that vinyl carboxylates are not connected via hydrogen bonding in the **1a/**toluene self-assemblies. Contrary to **1a**, the hydrogen-bounded carboxyl band at 1749 cm^−1^ of valine **2a** in toluene was shifted to 1757 cm^−1^ by the gel melting, suggesting that breaking central hydrogen bonds caused significant changes in the self-assembly and surrounding of the carboxylate groups. The presence of FTIR bands at 3385 cm^−1^ in **1a**/toluene gel—which correspond to non-hydrogen-bonded NH groups—can be explained by the specific self-assembly of the organogelator, in which all NH amide protons did not participate completely in hydrogen bonding, or by the existence of solvated molecules (Supplementary Materials Table S2). As previously noted, **1a** was a poor gelator of toluene and formed gel only through the application of ultrasound. Without sonification, only small, granular gels in the solvent were observed after 0.5–1 h. The FTIR investigation indicated the same organization in the gelling forms of **1a** in toluene in both methods of gel preparation (Figure S8a,b). The reproducibility of the gel-sol temperature transition was determined experimentally. After heating/cooling treatment of up to three cycles, no evidence of a chemical change was observed from FTIR spectra (Figures S18 and S19). From the obtained temperature dependent FTIR spectra of the **1a** and **2a**/toluene supramolecular gels, we concluded that molecules in the gels were crosslinking with multiple thermoreversible hydrogen bonds.

**Figure 3:**
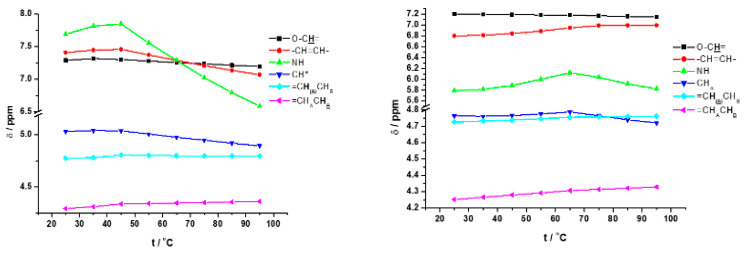
Chemical shifts of 1**a** (**left**) and (**b**) **2a** (**right**) protons in toluene-d_8_ gels in the temperature range of 25–95 °C.

The temperature-dependent ^1^H NMR spectra of the **2a**/toluene-d_8_ and **1a**/toluene-d_8_ gels showed a downfield shift of the NH protons before the gel melting temperatures (Tg) (Tg = 56 °C for **1a** and Tg = 75 °C for **2a**) and upfield shifts after the Tg as a result of destruction of the supramolecular self-assembly (Figure 3).

Associated with this process, in both toluene organogels the signals of asymmetric C*H and –O-CH= protons were shifted upfield, while those of =CH_A_H_B_ protons were shifted slightly downfield. The fumaryl –CH=CH- protons of **1a**/toluene and **2a**/toluene were shifted in opposite directions: in **1a**/toluene it was upfield, and in **2a**/toluene it was downfield (Figure 3).

**Figure 4:**
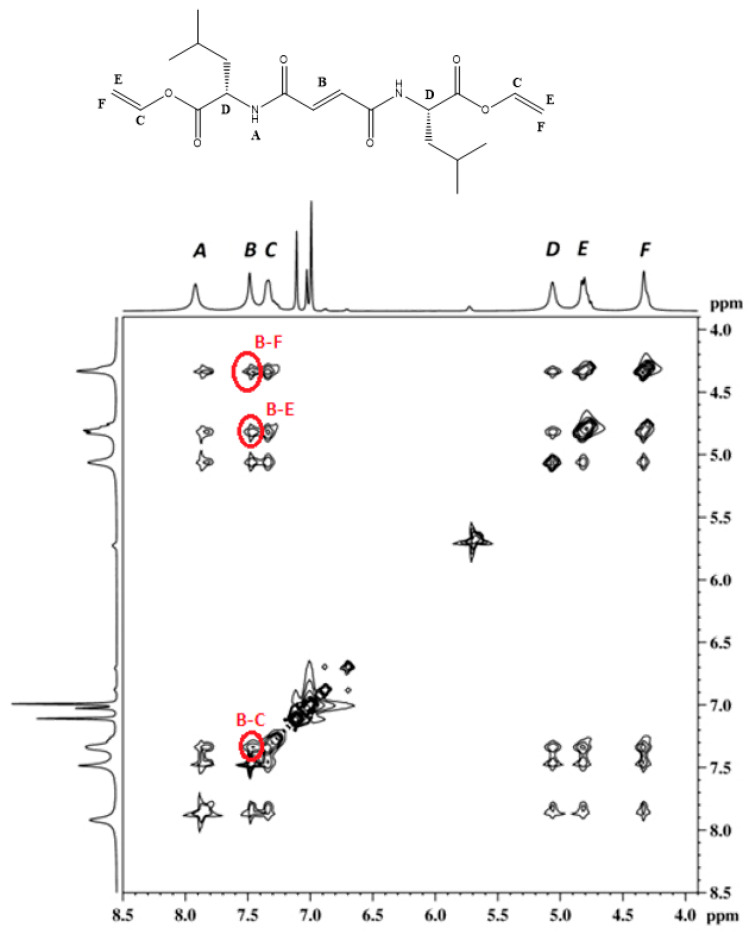
2D ^1^H NOESY spectra of **1a** toluene-d_8_ gel.

Recording and analyzing 2D NOESY spectra of gels (**1a** and **2a** toluene gel), we were able to gather additional information on the interactions between specific groups and atoms in the gel assemblies. The most important NOE interaction of note was the interaction between the fumaramide vinyl protons and the proton of the vinyl ester group (Figure 4 and Figure S9). This interaction was not possible intramolecularly, since the protons were too far apart. The models of gelator **1a** and **2a** obtained by a molecular modelling (conformational search and energy minimization) using Sybyl-X [72] indicated that the distance between the protons of the fumaric double bond and the protons vinyl ester group is approximately 8.7–9.7 Å in both cases (Figures S10 and S11). The above results obtained from the NOESY spectra indicated the intermolecular interaction between the molecules in the self-assembly. Furthermore, the proximity of the vinyl ester and fumaramide double-bond units was favorable to gamma-ray- and ultraviolet-assisted polymerization reactions. In addition, the 2D NOESY spectra of **2a**/toluene gel heated at 40 °C (thermal gel-to-sol phase transition) contained no intramolecular interaction between the fumaramide vinyl protons of neighboring molecules. After the gel-sol transition, the gelator molecules of **2a** were no longer included in the self-assembly (Figure S20).

**Figure 5:**
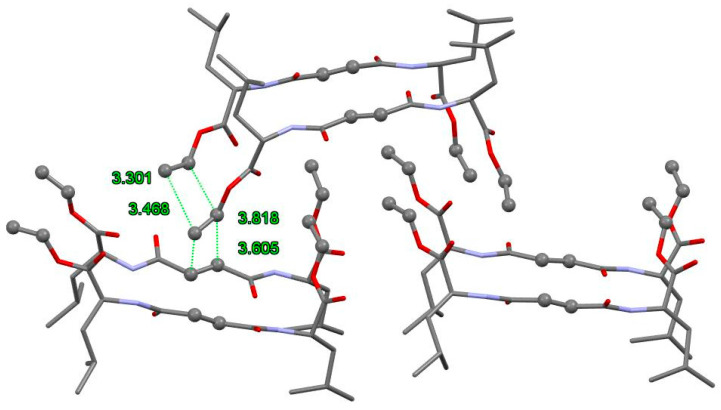
Favorable packing of the molecules **1a** for polymerization reactions obtained by molecular modelling. Reactive olefin carbons are represented in a ball-and-stick model. Hydrogen atoms are omitted for clarity.

Molecular modeling studies were performed using the Tripos force field as implemented in the SYBYL-X software suite [72]. All calculations were performed in the gas phase. Gasteiger–Hückel charges were used in all calculations. The compounds were optimized for the minimization of energies and geometry optimization, convergence criterion, and root-mean-square (RMS) gradient at 0.01 kcal/mol Å, and the iteration limit was set at 10,000, by considering the determined distance-dependent dielectric constant of 4.0. The strategy for performing the energy minimization of compounds **1a** and **2a** was selected in accordance with the literature parameters for protein molecular modelling [73]. It was found that through the dielectric constant, ε = 4 or 20, a distance-dependent dielectric function, and stepwise energy minimization, it was possible to reproduce X-ray structures very accurately without including explicit solvent molecules. The assembly of the group of six gelator molecules was generated from known crystal structures of methyl esters of bis(leucine)fumaramide (**2a**) [69] and determinate crystal structures bis(valine)fumaramide (**2b**), by changing methyl ester groups to vinyl. The molecular modelling and energy minimization of the group of six gelator molecules **1a** and **1b** yielded low energy conformations interconnected by hydrogen bonds (Figure 5, Figures S14 and S15). The molecular modeling studies of **1a** and **2a** revealed that in these compounds both the central amide carbonyl groups encountered severe steric repulsions with the neighboring molecule amide carbonyls, respectively. In the most stable conformation of these compounds, both amide groups were in the same plane vertical orientation to the double bond of fumaryl group. As a consequence, the hydrogen-bonding moieties were all oriented along a common axis and thus strongly favored the aggregation by hydrogen bonding in only one vertical dimension. During the minimization process, the gelator molecules accumulated in the self-assembly, enabling the required proximity of vinyl reactive groups for the polymerization reaction. Furthermore, the compact packing of self-assembled molecules was responsible for the stability of the gels of fumaramide derivates **1a** and **2a**.

Generated models of molecular self-assembly showed that reactive vinyl groups (the central vinyl groups of fumaramide and a vinyl ester) could be located at a distance from 3.5 up to 4 Å. Polymerizable groups located in the model (close enough and in favorable orientation) were able to form a covalent bond during gamma and ultraviolet irradiation, which was shown to be the necessary condition for a topochemical reaction in the solid-state [74]. Schmidt proposed that these reactions occur if the reacting groups attain a planar or near-planar orientation and are placed within an optimum distance of 3.5–4.2 Å in the crystal lattice [75].

### 3.4. Gamma-Ray- and Ultraviolet-Induced Polymerization of Gels ***1a*** and ***2a***

The influence of the supramolecular organization on the polymerization of fumaramide organogelators assembled in the gel network was investigated on **1a**/DMF-H_2_O, **1a**/toluene and **2a**/toluene gels.

Gamma irradiation of **1a**/DMF-H_2_O gel resulted in the formation of an insoluble solid material. There were no differences in the optical appearance of the initial gel and the polymer material. The gamma irradiation was carried out by the gamma rays using ^60^Co source at a total dose of 200 kGy and dose rate of 14 kGy/h. All samples were sealed before irradiation in a glass tube after argon bubbling and removing oxygen. The polymerized products were insoluble in common organic solvents. The product was washed with toluene and dried. Polymerization was complete, since no organogelator **1a** was detected in the supernatant. By the stepwise addition of the solvent to the dried polymer material, we showed that the polymer could immobilize approximately the same volume of the solvent as the parent organogel (tested with EtOH, which **1a** cannot gelatinize).

**Figure 6:**
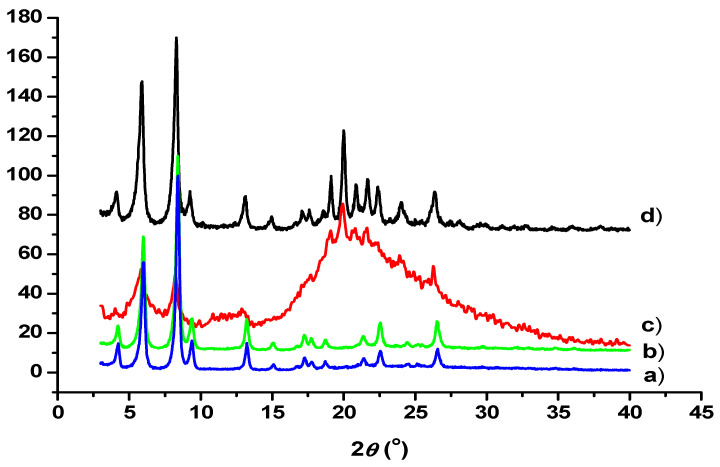
XRD pattern of dose-dependent polymerization of **1a/**DMF-H_2_O gel: (**a**) xerogel (0 kGy); (**b**) 30 kGy; (**c**) 50 kGy; (**d**) **1a** powder (crystalline solid).

X-ray diffraction (XRD) investigations showed amorphous properties in the new polymer material. A dose-dependent investigation (0–50 kGy) of **1a** polymerization by the amorphous material was observed, as seen in Figure 6. The gamma irradiation could be responsible for the formation of the amorphous material. As seen in Figure 6 (line c), after 50 kGy radiation dose exposures, amorphization was evident from the reduction in intensity and significant broadening of the diffraction lines as well as the appearance of the scattering halo, i.e., the broad hump in the pattern centered at ca. 20 degrees. Since all the diffraction peaks were losing intensity and broadening, we concluded that the amorphization upon irradiation affected the whole of the 3D lattice and all of the reflections. The degree of polymerization was dependent on the applied dose of the irradiation.

**Figure 7:**
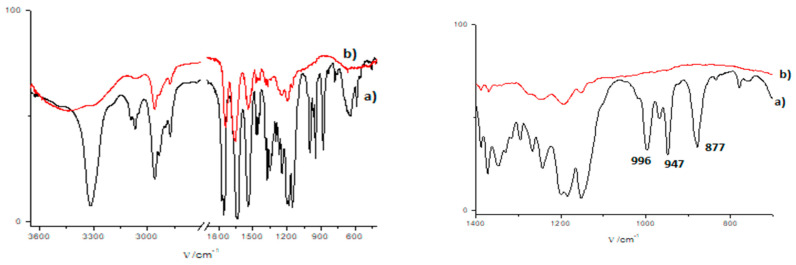
FTIR spectra of **1a** (red) and the product of **1a**/(DMF-H_2_O gel (black) gamma polymerization. Total spectra (**left**) and the zoom-in spectra in the range between 1400 and 700 cm^−1^ (**right**).

It is interesting to note that only a small quantity of fiber bundles was observed in the polymer network. This observation could indicate that the fiber bundling observed in the gel network TEM was mostly associated with the drying of the gel material for the preparation sample for microscopic investigations. The **1a**/DMF-H_2_O gel was placed onto an open glass plate and exposed to gamma irradiation; the resulting polymer was consistent with XRD, suggesting that there were no changes in the unpolymerized organogelators’ self-assembly during the process of polymerization due to the total administered dose of 50 kGy. At the total dosage of 50 kGy, when most of the organogelator was polymerized, a self-organization similar to those of crystalline organogelators was observed. Additionally, a quantity of microspheres in approximately the same quantity as the mixture of fiber bundles and single fibers were observed (Figures S2–S6). The product of the polymerization was insoluble in common organic solvents; therefore, it was washed with toluene and methanol and dried. FTIR spectra of the product of **1a**/(DMF-H_2_O gel after gamma polymerization indicated a complete disappearance of the peaks at 996 cm^−1^ ascribed to the fumaryl (C=C) double bond and peaks at 947 cm^−1^ characteristic of vinyl esters. That suggested that both the reactive groups were involved in the polymerization of vinyl ester bis(L-leu)fumaramide (**1a**) (Figure 7).

**Figure 8:**
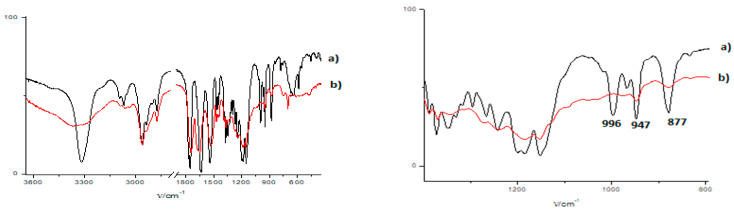
FTIR spectra of **1a** (black) and the product of **1a**/(DMF-H_2_O gel (red) ultraviolet polymerization Total spectra (**left**) and the zoom-in spectra in the range between 1400 and 800 cm^−1^ (**right**).

Polymerization studies using ultraviolet radiation were conducted in a quartz cuvette with a high-pressure mercury lamp (100 W) with 5 mol% benzophenone photo = initiator (paper) as a starter of reaction; the reaction time was 10 h. The experiments were performed on **1a**/DMF-H_2_O, **1a**/toluene, **2a**/toluene gels, and leucine methyl ester (**1b)** derivate in toluene solution. The product of the polymerization was insoluble in common organic solvents; therefore, it was washed with toluene and methanol and dried. As the result of the polymerization, peaks in the polymer FTIR spectra characteristic of C=C-H stretching at 3093, 3068, and 3050 cm^−1^ and the peaks ascribed to the deformation of the ethylene moiety at 996 cm^−1^ (ascribed to fumaryl) and 947 cm^−1^ (characteristic of vinyl esters) were disappeared (Figure 8). This suggested that both the reactive groups were involved in the reaction. UV polymerization of bis(L-leu)fumaramide methyl ester (**2a**) gave (2 + 2) cycloadditions with and without the photo-initiator (Figure 8 and Figure S1).

### 3.5. TEM and SEM Investigations

**Figure 9:**
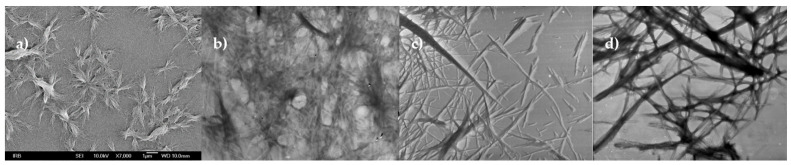
(**a**) SEM micrograph of freshly prepared **1a**/DMF-H_2_O gel (**b**) micrograph of **1a**/DMF-H_2_O gel network; freshly prepared gel negatively stained with PWK; (**c**) freshly prepared gel shaded with Pd; and (**d**) TEM micrograph of 20 h aged 1a/DMF-H2O gel.

Leucine fumaramide **1a** had a tendency to form spherulite-like gel networks in a polar DMF/H_2_O solvent mixture. The transmission electron microscopy (TEM) investigation provided detailed information on the self-assembly of **1a** and showed that the gel network consisted mostly of fibers. The presence of large fiber bundles and tapes or twisted fiber or tapes can also be seen in Figure 9b–d. The gel was turbid and stable during the investigations.

**Figure 10:**
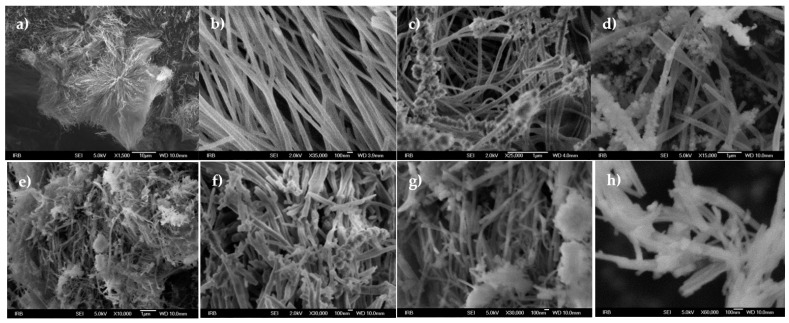
(**a**–**d**) SEM micrograph of polymer network obtained from **1a**/DMF/H_2_O gel after gamma ray radiation; (**e**–**h**) SEM micrograph of polymer network obtained from **2a**/DMF/H_2_O gel after UV rays-radiation.

Morphological investigations by scanning electron microscopy (SEM) showed that the morphology of the gel network did not change significantly upon polymerization. Figure 10a–h confirmed the polymerization of the self-organized aggregates. All morphological forms (fibers, bundled fibers, twisted fibers, and tapes) present in the gel network were also part of the polymer network, but some quantity of microspheres was present in approximately the same quantity as the mixture of fiber bundles and single fibers, as shown in Figures S2–S6.

## 4. Conclusions

We showed that vinyl esters of bis(leucine or valine)fumaramides (**1a**, **2**a) exhibited moderate gelling properties of the solvents with different polarities in forming stable thermoreversible gels. Morphology studies by electron microscopy (TEM and SEM) showed that the gels contained a filamentous structure composed of fibers and fiber bundles of nanometer dimensions entangled or organized in a three-dimensional network. Leucine and valine fumaramide derivates (**1a**, **2a**) differed by only one methylene group at the asymmetric center but possessed different gelator properties. Spectroscopic investigations (^1^H NMR, NOESY, and FTIR) showed that self-assemblies of leucine and valine were organized in a very similar way, i.e., through hydrogen bonding with central fumaramide groups as the major binding interaction between molecules. Secondary noncovalent binding interactions in self-assemblies were achieved through lipophilic interactions of amino acid side chains and via interactions of vinyl ester groups. The crystal structure confirmed that bis(leucine)fumaramide (**1b**) formed layers of molecules connected by hydrogen bonds that were oriented parallel to each other, unlike **2b*,*** where the layers formed a herringbone pattern. Based on the crystal structures of methyl ester bis(leucine or valine)fumaramides (**1b**, **2b**), model assemblies of six vinyl esters of bis(leucine or valine)fumaramides (**1a** and **2a**) were generated, connected by H bonds. Molecular modeling studies, including the energy minimization assembly of a group of six gelator molecules (**1a** and **2a**) interconnected by hydrogen bonds, showed that reactive vinyl group (the central vinyl group from fumaryl and a vinyl ester) molecules were located at a distance of 3.5 to 4 Å, which is the required condition for the implementation of a topochemical reaction in the solid-state. Furthermore, this property was responsible for the somewhat greater stability of the gels based on leucine derivate **1a**. In both cases, in the self-assemblies of leucine and valine fumaramide (**1a**, **2a**) the reactive vinyl groups were sufficiently close and favorably oriented to form covalent bonds upon gamma- and ultraviolet-radiation-initiated polymerization. The formation of polymeric materials was proved by FTIR spectroscopy. During the polymerization of 1a/DMF-H_2_O gel, the morphological properties of the gel network were preserved in the polymer network. An advantage of the described polymeric material was the ability to immobilize the solvent by swelling. This approach to the polymerization of fumaramides can be extended to the preparation of various new functional materials with improved properties.

## Figures and Tables

**Table 1:** Gelation efficiencies expressed as maximal volume (ν_max_) immobilized by 10 mg organogelators.

Solvent	1a	2a
H_2_O	ns	ns.
H_2_O/DMSO	ng	6.2 + s5.3 *
H_2_O/DMF	0.44 + 0.48 *	cryst.
EtOH	sol.	cryst.
±2-octanol	sol.	cryst.
THF	0.26 + 2.8 **	sol.
dioxane	sol.	sol.
acetone	0.1 + 1.3 **US	cryst.
EtOAc	0.2 + 1.2 **US	cryst.
CH_2_Cl_2_	ng	sol.
CH_3_CN	cryst.	cryst.
toluene	0.2 *US*	1.6 *US*

ns = not or low soluble; * = DMSO; ** = hexane; *US* = ultrasonification: sol. = solution; ng = not gel; cryst. = crystals.

## Supplementary Materials

The following are available online at https://www.mdpi.com/article/10.3390/polym14010214/s1. ^1^H-NMR, ^13^C-NMR, HRMS spectra and FTIR data for compounds: **1a** and **2a**; Synthetic procedure, NMR spectra and data, HRMS spectra for **1b**, and crystallographic data for **2b** (CCDC: 2124266); Figure S1. MS scan UV polymerization reaction of bis (L-leu)fumaramide methyl ester **1b**; Scanning Electron Microscope (SEM) Investigation, Figures S2–S4; SEM micrography form obtained by gamma rays polymerization **1a**; Figures S3–S6 SEM micrography of UV rays polymerization **1a**; Figure S7. SEM micrography of UV polymerization CH_2_Cl_2_ solution of compound **1a**; FTIR Measurements: Table S2. Characteristic FTIR bands (cm^−1^) of **1a**, **2a** (gel, crystal, product gamma, irradiation in solution), Figure S8. Temperature dependent FTIR spectra of (a); **1a**/toluene gel; 2D NOESY spectra: Figure S9 NMR; Selected region of 2D NOESY spectra of (a) **1a**/toluene gel; (b) **2a**/toluene gel; (c) **1a**/DMF-H_2_O gel; Figure S10. Distances H–H (vinyl ester:vinyl fum) in fully minimized the lowest energy conformations of **1a**, Figure S11**.** Distances H–H (vinyl ester:vinyl fum) in fully minimized the lowest energy conformations of **2a**; Molecular modelling: Figure S12**.** Crystal structure of **1b** [71], Figure S13. Crystal structure **2b** (CCDC: 2124266), Figure S14**.** (a) Distances reactive groups (C=C) under 4 Å in the model of favorable packing of the 6 molecules linked by amide H-bonds (NH-O=C) (**1a**) obtained by molecular modelling. Figure S15**.** Distances reactive groups (C=C) ~4 Å in the model of favorable packing of the 6 molecules linked by amide H-bonds (NH-O=C) (**2a**) obtained by molecular modelling., Figure S16**.** Hydrogen bond pattern in crystal structure of (**1b**) [71]. Figure S17**.** Hydrogen bond pattern in crystal structure of (**2b**) CCDC: 2124266; Figure S18 Temperature FTIR spectra **1a**/toluene-d_8_ (c = 0.23 M), Figure S19. Temperature FTIR spectra **2a**/toluene-d_8_, and Figure S20. Selected region of 2D NOESY spectra of **2a**/toluene gel at 40 °C are available in Supplementary data. Supplementary data in the form of a CIF have been deposited with the Cambridge Crystallographic Data Centre (CCDC: 2124266 for **2b**).
