# Supplementary material for: Gamma Radiation- and Ultraviolet-Induced Polymerization of Bis(amino acid)fumaramide Gel Assemblies
Source: Polymers (Basel). 2022 Jan 5;14(1):214. doi: 10.3390/polym14010214 (PMC8747669; doi:10.3390/polym14010214)
Supplement: Supplementary file 1 [file polymers-14-00214-s001.zip › polymers-1520027-supplementary.pdf]

Electronic Supplementary Information for:

Gamma Radiation- and Ultraviolet-induced Polymerization of Bis(amino acid)fumaramide Gel Assemblies

Tomislav Gregorić [1], Janja Makarević [1], Zoran Šrefanić,[2] Mladen Žinić [1,3], and Leo Frkanec [1,*]

[1]Laboratory of Supramolecular Chemistry, Division of Organic Chemistry and Biochemistry, Ruđer Bošković Institute, Bijenička c. 54, 10000 Zagreb, Croatia; tgregor@irb.hr

[2]Laboratory for chemical and biological crystallography, Division of Physical Chemistry, Ruđer Bošković Institute, Bijenička c. 54, 10000 Zagreb, Croatia, Zoran.Stefanic@irb.hr

[3]Croatian Academy of Sciences and Arts, Trg Nikole Šubića Zrinskog 11, HR-10000 Zagreb, Croatia; zinic@hazu.hr

*****Correspondence: frkanec@irb.hr; Tel.: +385 1 468 0217

Table of content:

1. **^{1}H-NMR, ^{13}C-NMR, HRMS spectra and FTIR data for compounds 1a** and **2a**
2. **Synthetic procedure**, NMR spectra and data, HRMS spectra for **1b**, and crystallographic data for **2b (CCDC: 2124266)**

Figure S1: MS scan UV polymerization reaction of bis (L-Leu) fumaramid methyl ester. Product of [2+2] cycloaddition m/z [M+H]+: $C_{36}H_{60}N_4O_{12}$, calculate: 741,42, found: 741,2.

Scanning Electron Microscope (SEM) Investigation.

Figure S2 SEM micrography form obtained by gamma rays polymerization of **1a**/DMF-H_2O gel network

Figure S3; SEM micrography of gamma rays polymerization of **1a**/DMF-H_2O gel network on a glass slice

Figure S4; SEM micrography of product from the gamma rays polymerization **1a**/toluene gel network

Figure S5; SEM micrography of UV-rays polymerization from **1a**/DMF-H_2O gel network.

Figure S6; SEM micrography of UV rays polymerization from **2a**/ toluene gel network

Figure S7: SEM micrography of UV polymerization CH_2Cl_2 solution of compound **1a**

3. **FTIR Measurements**

Table S2; Characteristic FTIR bands (cm^{-1}) of **1a**, **2a** (gel, crystal, product gamma, irradiation in solution).

Figure S8. Temperature dependent FTIR spectra of a); **1a**/toluene gel (ultrasound induced gelation) b) **1a**/toluene-d_8 gels (without application of ultrasound) in the temperature range of 20-95 °C and c) **1a**/toluene gel with 5 % mol benzophenone in the temperature range of 20-100 °C

4. 2D NOESY spectra

Figure S9 NMR; Selected region of 2D NOESY spectra of a) **1a**/toluene gel; b) **2a**/toluene gel; c) **1a**/DMF-H_2O gel; [***A***=NH, ***B*** **<u>CH</u>**=**<u>CH</u>**(fum), ***C*** O-**<u>CH</u>**=$CH_{2, viny}$, ***D*** CH*, ***E*** and ***F*** O-CH=**<u>CH</u>**$_{\mathbf{2}, vinyl}$ protons.

Figure S10. Distances H – H (vinyl ester : vinyl fum) in fully minimized the lowest energy conformations of **1a** (**C – B:** 6.6 – 6.8 Å; **E – B:** 5.6 – 6.7 Å;**F – B** 7.0 - 7.7 Å)

Figure S11. Distances H – H (vinyl ester : vinyl fum) in fully minimized the lowest energy conformations of **2a** (**C – B** 6.6 – 7.3 Å; **E – B** 6.0 – 6.3 Å; **F – B** 7.2 - 7.5 Å)

5. Molecular modelling

Figure S12. Crystal structure of *N,N'*-bis[(2*S*)-1-methoxy-3-methyl-1-oxobutane-2-yl] fumaramide (**1b**); a bilayer of molecules linked by H-bonds (1D Fum NH – O=C); 3D all *i*-Bu groups (R-Leu) are oriented are oriented towards each other, OMe groups are located on the other side of the bilayer[71].

Figure S13. Crystal structure *N,N'*-bis[(2S)-1-methoxy-4-methyl-1-oxopentane-2-yl] fumaramide (**2b**) (1D H-bonds Fum NH – O=C); the layers are vertically oriented like a "herringbone" 3D *i*-Pr group (R-Val) together with OVin oriented in a "cavity", OMe groups oriented towards to another OMe (CCDC: 2124266).

Figure S14. a) Distances reactive groups (C=C) under 4 Å in the model of favourable packing of the 6 molecules linked by amide H-bonds (NH-O = C) (**1a**) obtained by molecular modelling. Hydrogen atoms are omitted for clarity. b) enlarged part of the structure.

Figure S15. Distances reactive groups (C=C) ~ 4 Å in the model of favourable packing of the 6 molecules linked by amide H-bonds (NH-O = C) (**2a**) obtained by molecular modelling. Hydrogen atoms are omitted for clarity. b) enlarged part of the structure.

Figure S16. Hydrogen bond pattern in crystal structure of *N,N'*-bis[(2*S*)-1-methoxy-3-methyl-1-oxobutane-2-yl] fumaramide (**1b**).[71]

Figure S17. Hydrogen bond pattern in crystal structure of *N,N'*-bis[(2S)-1-methoxy-4-methyl-1-oxopentane-2-yl] fumaramide (**2b)** CCDC: 2124266.

Figure S18 Temperature FTIR spectra **1a**/toluene-d_8 (c= 0.23 M) before heating (black) at 100 °C (red) and after cooling (blue)

Figure S19 Temperature FTIR spectra **2a**/toluene-d_8 (c=4.2x10^{-2} M) gels) before heating (black) at 100 °C (red) and after cooling (blue)

Figure S20. Selected region of 2D NOESY spectra of **2a**/toluene gel at 40 °C; [***A***=NH, ***B*** **<u>CH</u>**=**<u>CH</u>**(fum), ***C*** O-**<u>CH</u>**=$CH_{2, viny}$, ***D*** CH*, ***E*** and ***F*** O-CH=**<u>CH</u>**$_{\mathbf{2}, vinyl}$ protons

1. **^{1}H-NMR, ^{13}C-NMR, HRMS spectra and FTIR data** for compounds **1a** and **2a**

***N,N'*-bis[(2S)-1-vinyloxy-4-methyl-1-oxopentane-2-yl] fumaramide (1a)**

White powder 221 mg (56 %, yield) M.p.=177 - 179 °C (CH_3CN), $[\alpha]_D$ = - 54,5 (γ = 1 g/mL, MeOH); ^{1}H NMR (600 MHz, DMSO-d$_6$, 20 °C): δ = 8.95 (2H, d, *J*= 6 Hz NH), 7.21 (2 H, dd, *J*=6.2, *J*=13.9, OC**H**=CH$_2$), 6.92 (2H, s, HC=CH), 4.96 (2H, dd, *J*=1.9, *J*=13.9, OCH=C**H$_A$**H$_B$), 4.73 (2H, dd, *J*=1.9, *J*=6.2, OCH=CH$_A$**H$_B$**), 4.43-4.39 (2 H, m, CH$_\alpha$), 1.67-1.56 (6 H, m, CH$_{2,\beta}$ and CH$_\gamma$), 0.92 (6H, dd, *J*=6.2, CH$_{3,\delta}$), 0.87 (6H, dd, *J*=6.2, CH$_{3,\delta}$) ppm. ^{13}C NMR (75 MHz, DMSO-d$_6$, 20 °C): δ =170.1 (COO), 164.3 (CON), 141.7 (O**C**H=CH$_2$) 132.9 (HC=CH), 99.6 (OCH=**C**H$_2$), 51.0 (CH$_\alpha$), 39.6 (CH$_{2,\beta}$), 24.8 (CH$_\gamma$), 23.1, 21.6 (CH$_{3,\delta}$) ppm; FTIR (KBr) ν_{max}/cm^{-1} = 3320 (NH), 1755 (OC=O), 1630 (HNC=O, amide I), 1538 (HNC=O, amide II)

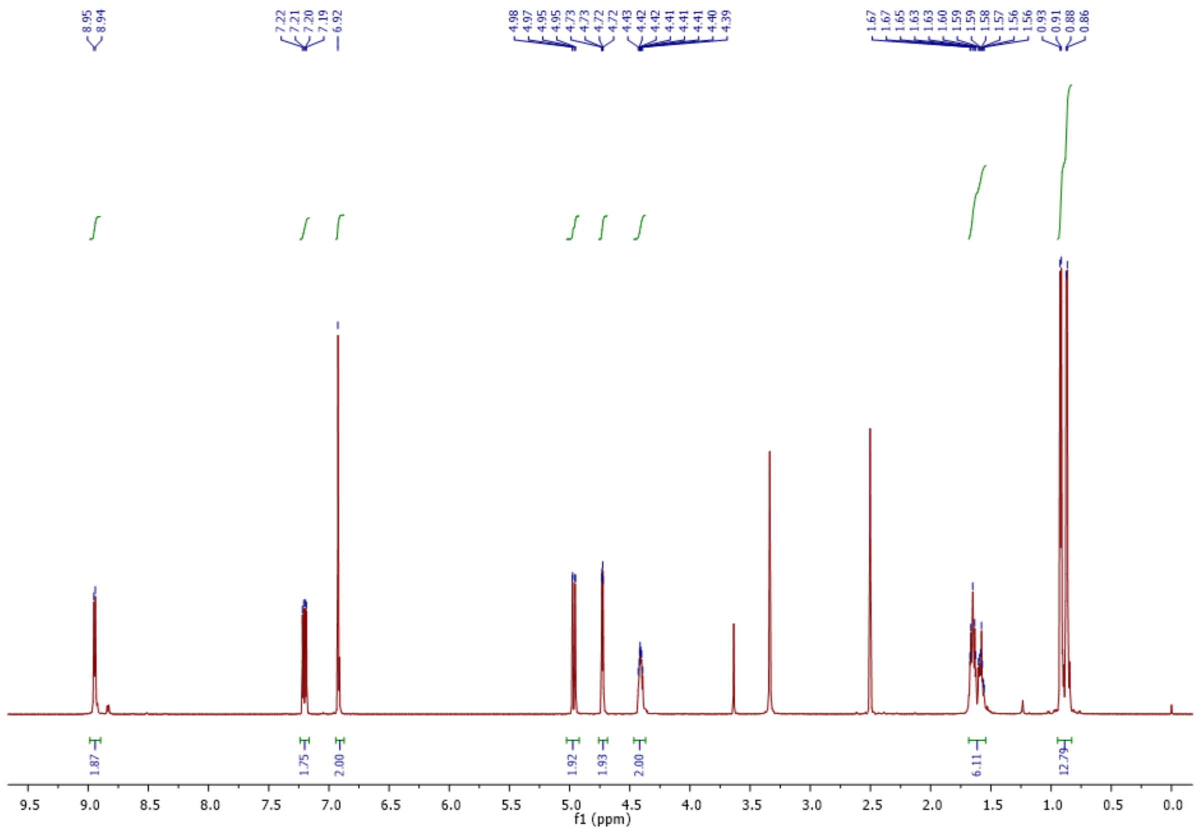

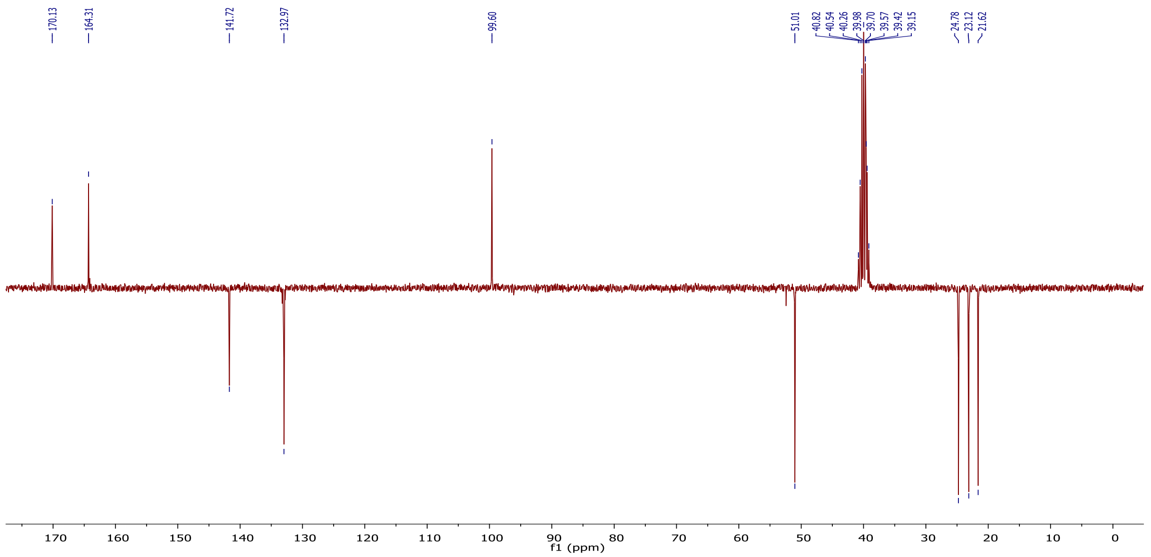
170.13
164.31
141.72
132.97
99.60
51.01
40.82
40.54
40.26
39.98
39.70
39.57
39.42
39.15
24.78
23.12
21.62
f1 (ppm)

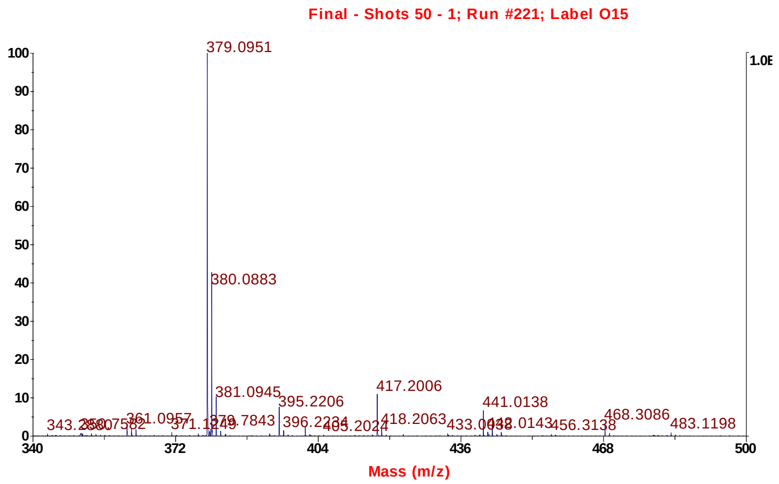
Final - Shots 50 - 1; Run #221; Label O15
379.0951
380.0883
381.0945
395.2206
417.2006
441.0138
468.3086
483.1198
Mass (m/z)

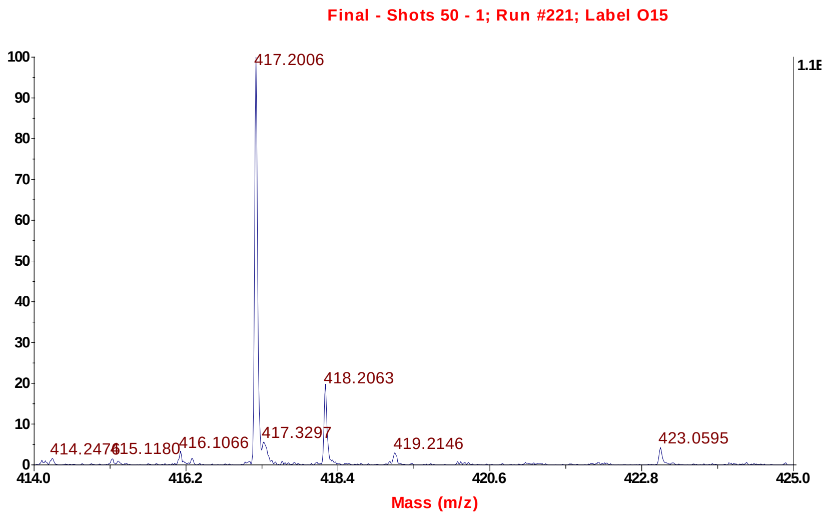

HRMS: m/z $[M+Na]^+$: $C_{20}H_{30}N_2O_6$, calculate: 417.2002, found: 417.2006.

N,*N*'-bis[(2*S*)-1-vinyloxy-3-methyl-1-oxobutane-2-yl] fumaramide **(2a)**

White powder 205 mg (55.9 %, yield), MP=175–176 °C (CH_3CN), $[\alpha]_D$ = -68.5 (γ = 1 g/mL, MeOH);

^{1}H NMR (300 MHz, $CDCl_3$, 20 °C): δ =7.24 (2 H, dd, *J*=6.2 Hz, *J*=13.9 Hz, OC**H**=CH_2), 7.1 (2H, s, HC=CH), 6.91 (2 H, d, *J*= 9 Hz, NH), 4.95 (2H, dd, *J*=1.7, *J*=13.9, OCH=C$\mathbf{H_A}$$H_B$), 4.79 (2 H, dd, *J*= 5.0 Hz, *J*= 9.0 Hz, CH_α), 4.65 (2H, dd, *J*= 1.8 Hz, *J*= 6.2 Hz, OCH=$CH_A$$\mathbf{H_B}$), 2.32-2.25 (2 H, m, CH_β), 1.00 (6H, 2d, *J*= 6.9 Hz, $CH_{3,\delta}$); 0.97 (6H, 2d, *J*= 6.9 Hz, $CH_{3,\delta}$) ppm. ^{13}C NMR (75.5 MHz, $CDCl_3$, 20 °C): δ =168.9 (COO), 164.8 (CON), 140.8 (O**C**H=CH_2) 133.3 (HC=CH), 99.0 (OCH=**C**H_2), 57.1 (CH_α), 31.4 ($CH_{2,\beta}$), 18.9 ($CH_{3,\gamma}$), 17.7 ($CH_{3,\gamma}$) ppm.; FTIR (KBr) ν_{max}/cm^{-1} = 3267 (NH), 1750 (OC=O), 1637 (HNC=O, amide I), 1553 (HNC=O, amide II)

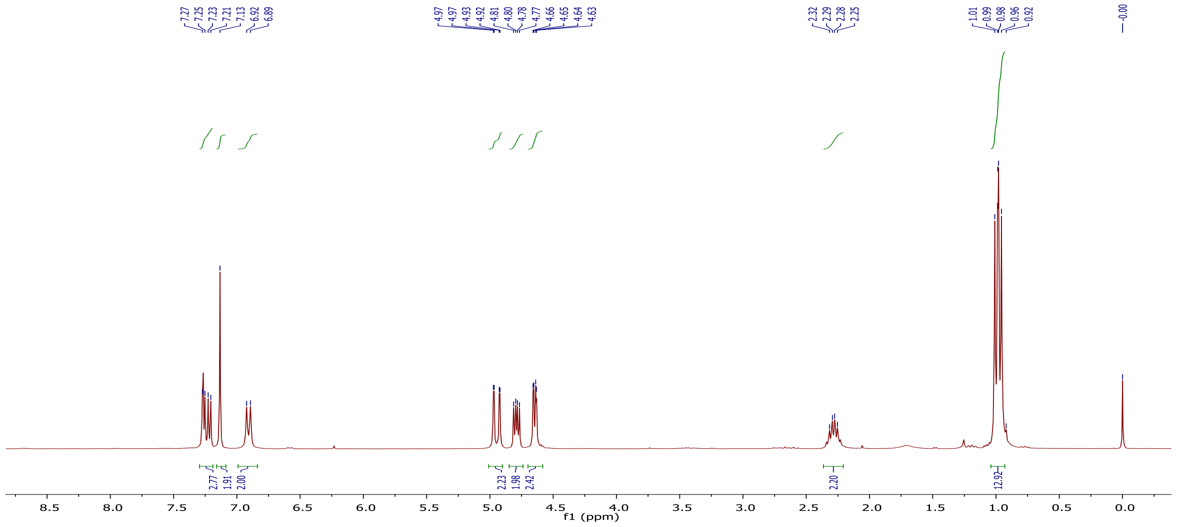

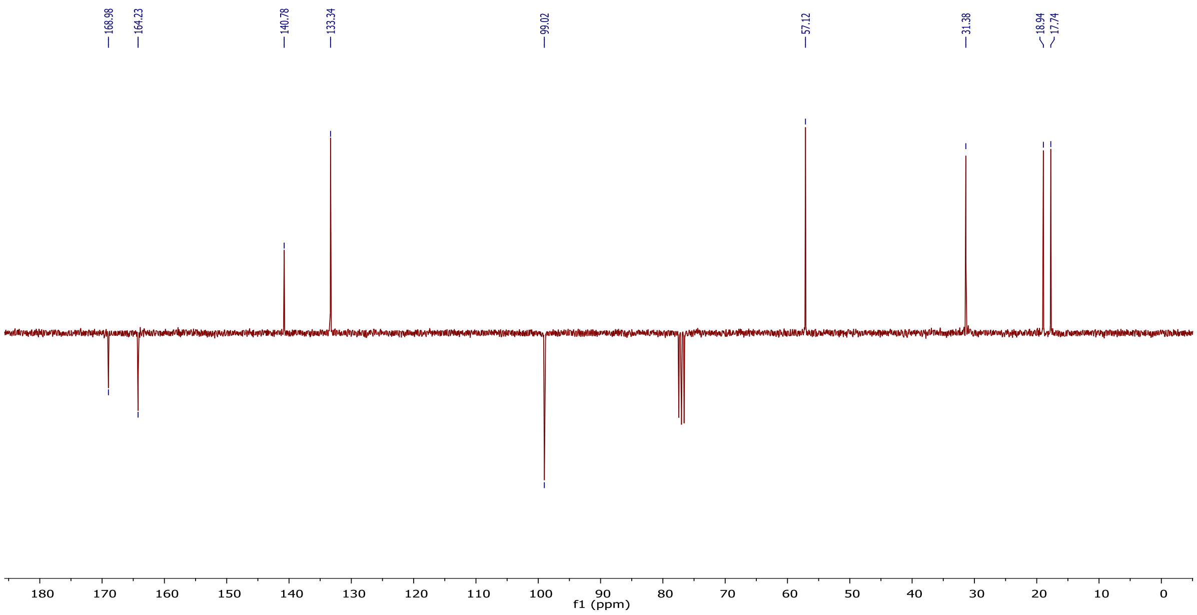
168.98
164.23
140.78
133.34
99.02
57.12
31.38
18.94
17.74
f1 (ppm)

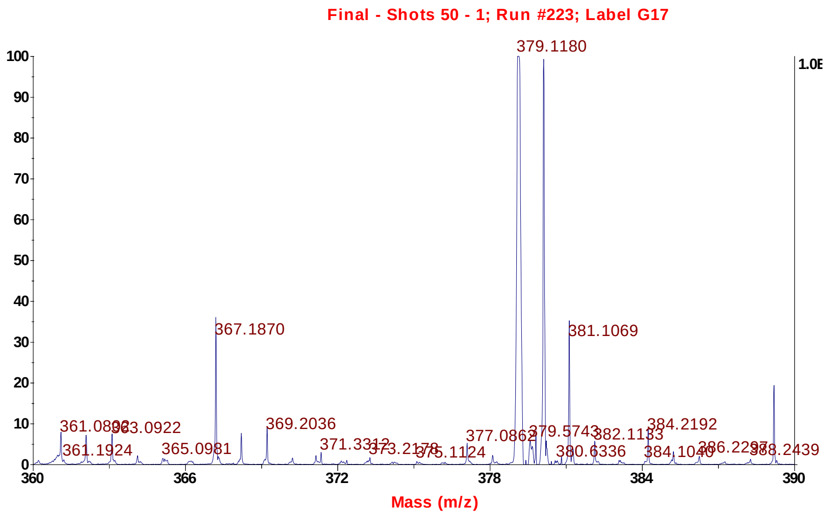
Final - Shots 50 - 1; Run #223; Label G17
379.1180
367.1870
381.1069
Mass (m/z)

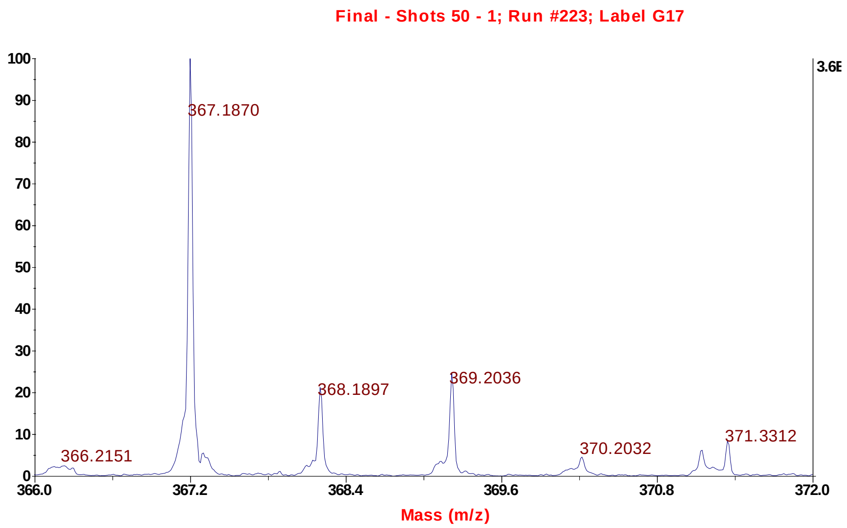

HRMS: *m/z* $[M+H]^+$: $C_{18}H_{26}N_2O_6$, calculate: 367.1869, found: 367.1870.

2 Synthetic procedure for methyl ester bis(Leu and Val) fumaramides (**1b** and **2b**)

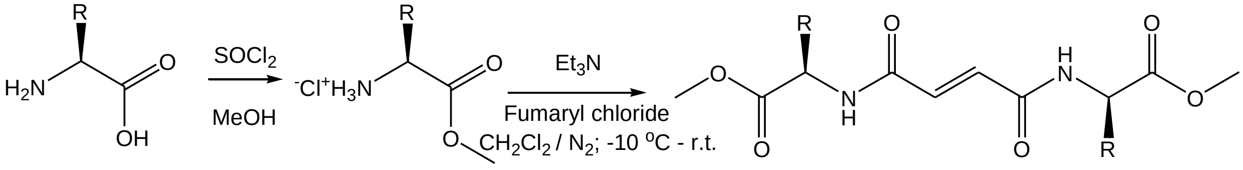

Scheme S1. Synthesis of bis(amino acid)formamide methyl esters **1b** and **2b.**

N,N'-bis[(2S)-1-methoxy-3-methyl-1-oxobutane-2-yl] fumaramide (**1b**)[69]

Compound 1b was synthesized according to general procedure. White powder 123.9 mg was obtained in a yield of 45.7 %. The NMR spectra of the obtained product correspond to the data from the literature.[69]

^{1}H NMR (300 MHz, DMSO-d$_6$): δ = 8.80 (2H, d, NH, J= 7.7 Hz), 6.90 (2H, s, HC=CH), 4.41-4.34 (2H, m, CHα), 3.32 (6H, s, OCH3) 1.63-1.52 (2H, m, CHβ i CH2), 0.87 (12H, dd, CH3, J= 6.2 Hz, J= 14.5 Hz) ppm; ^{13}C NMR (75 MHz, DMSO-d$_6$): δ = 172.6 (C=O), 163.7 (NHC=O), 132.5 (HC=CH), 51.9 (CHα), 50.5 (OCH3), 39.8 (CH2), 29.6 (CHβ), 22.6 (CH3), 21.1 (CH3) ppm.; FTIR (KBr) vmax/cm^{-1} = 3300 (NH), 1751 (OC=O), 1732 (OC=O), 1633 (HNC=O, amide I), 1534 (HNC=O, amide II).

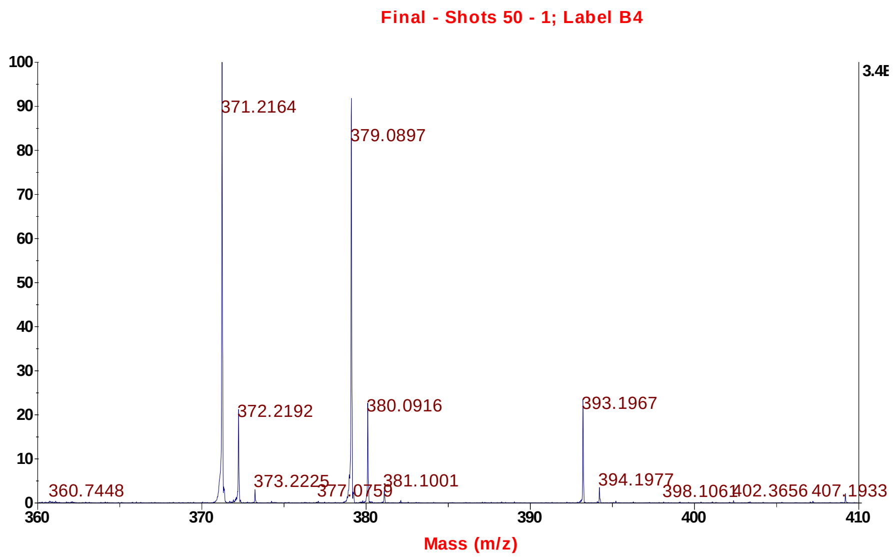

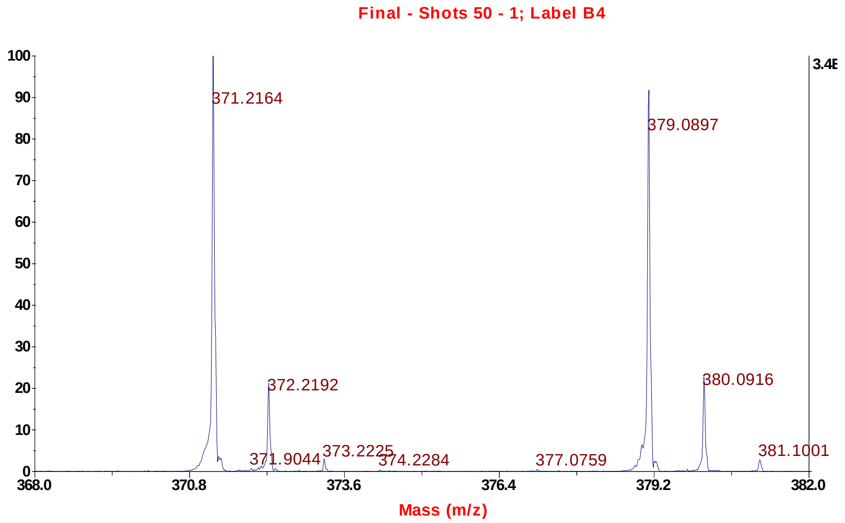

HRMS: m/z $[M+H]^+$: $C_{18}H_{30}N_2O_6$, calculate: 371.2182, found: 371.2164.

N,N'-bis[(2S)-1-methoxy-4-methyl-1-oxopentane-2-yl] fumaramide (**2b**)[70]

Compound **2b** was synthesized according to general procedure starting from L-Valine (234 mg, 2 mmol). White powder 125 mg was obtained in a yield of 53.5 %. The spectral data were consistent with those reported in the literature. [70]. Single crystal **1b** for X-ray diffraction analysis was obtained by recrystallization by slow evaporation from methanol (MeOH). Crystallographic data of **2b** have been deposited in the Cambridge Crystallographic Data Center under accession number **CCDC: 2124266.**

Crystallographic data for: *N,N'*-bis[(2S)-1-methoxy-4-methyl-1-oxopentane-2-yl] fumaramide **2b**

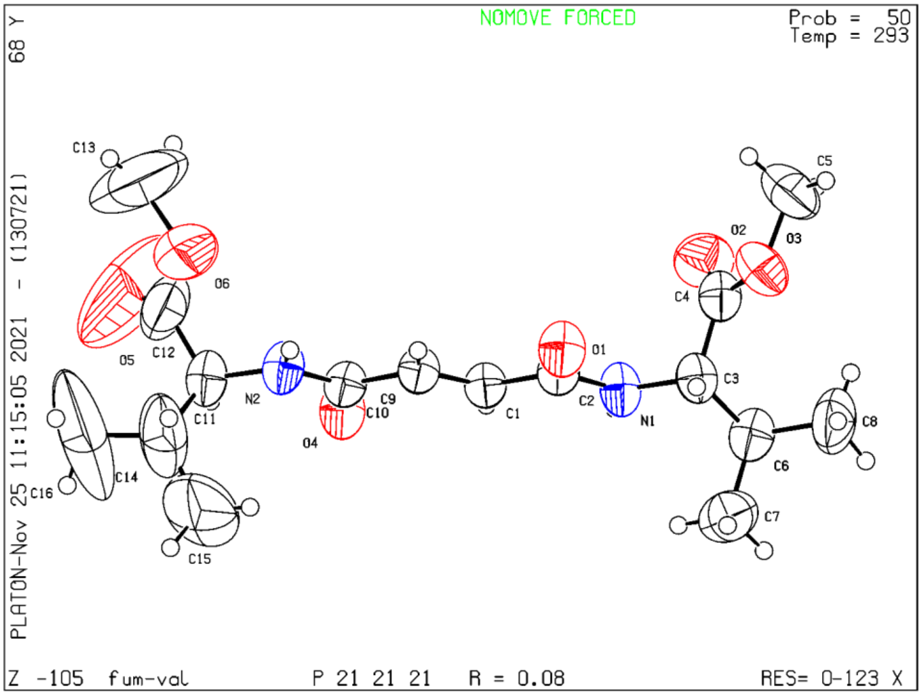

Figure CD1. Structure of title compound shown with probability ellipsoids at 50% level. It is visible that one side is highly disordered, while the other is completely ordered.

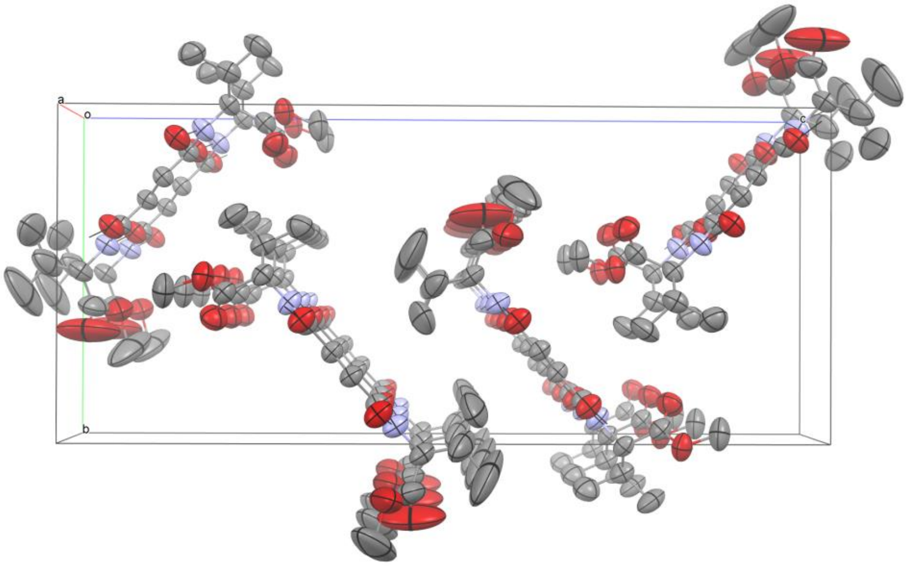

Figure CD2. The packing of molecules in the direction of crystallographic b axis. Molecules are connected by N-H...O hydrogen bonds in the direction of crystallographic b axis.

Table CD1. Crystallographic data collection and refinement data for the structure (*CCDC: 2124266*).

Formula	$C_{16}\ H_{26}\ N_2\ O_6$
Formula Weight	342.35
Crystal System	orthorhombic
Space group	$P2_12_12_1$
a, b, c [Å]	4.9093(3) 13.1211(12) 30.031(3)
V [Å^3]	1934.5(3)
Z	4
D(calc) [g/cm^3]	1.176
Mu(CuKa) [mm^{-1}]	0.750
F(000)	736
Crystal Size [mm]	0.05 x 0.1 x 0.2
**Data Collection**	
Temperature (°K)	293
Radiation [Å]	CuKα 1.54184
Theta Min-Max [°]	2.9, 76.0
Dataset	-3: 6 ; -14: 16 ; -37: 34
Tot., Uniq. Data, R_{int}	5949, 3646, 0.025
Observed data [I > 2.0 sigma(I)]	2695
**Refinement**	
N_{ref}, N_{par}	3646, 224
R, *wR*^{2}, *S*	0.0781, 0.2557, 1.07
Flack x	0.3(2)
Min. and Max. Resd. Dens. [e/Å^3]	-0.22, 0.52

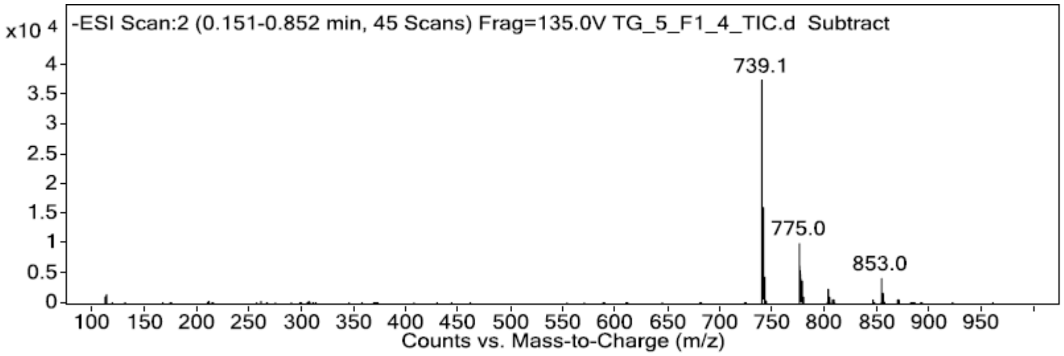

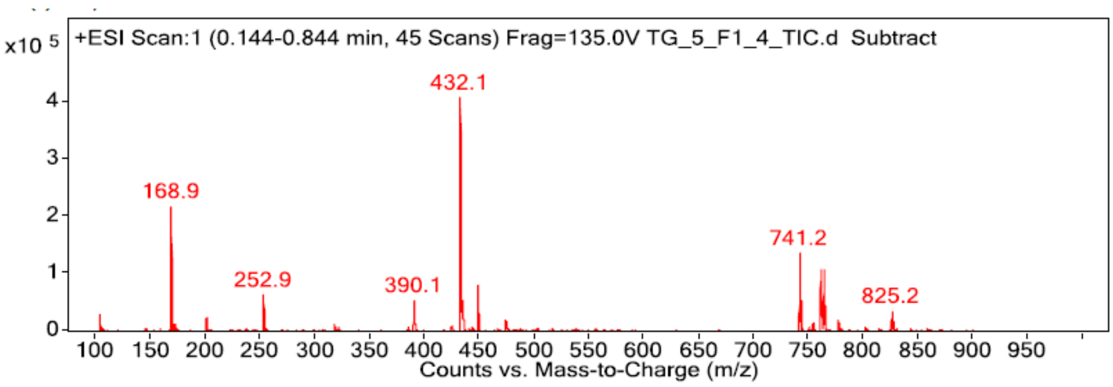

Figure S1: MS scan of UV polymerization reaction of bis (L-Leu) fumaramide methyl ester (**2**a). Product of [2+2] cycloaddition m/z [M+H]+: $C_{36}H_{60}N_4O_{12}$, calculate: 741.42, found: 741.2.

3. **Scanning Electron Microscope (SEM) Investigation**.

JEOL scanning electron microscope (SEM, Model JSM 5500LV) was used to study the fractured surface of the samples. The samples were first dipped into liquid nitrogen and snapped to half to prepare the fractured surfaces. Then, samples were mounted on the sample stub and were sputtered with gold. Property Testing of the produced composites was conducted in.

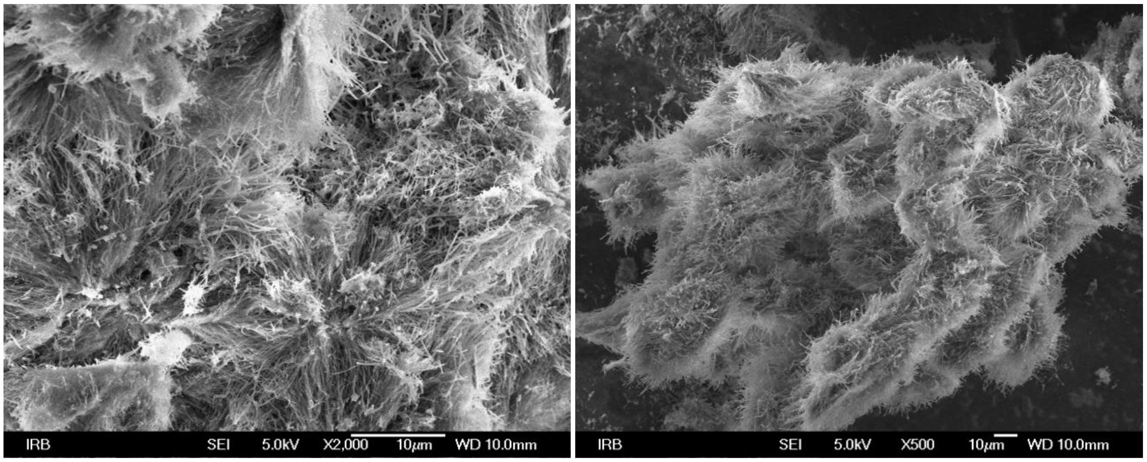

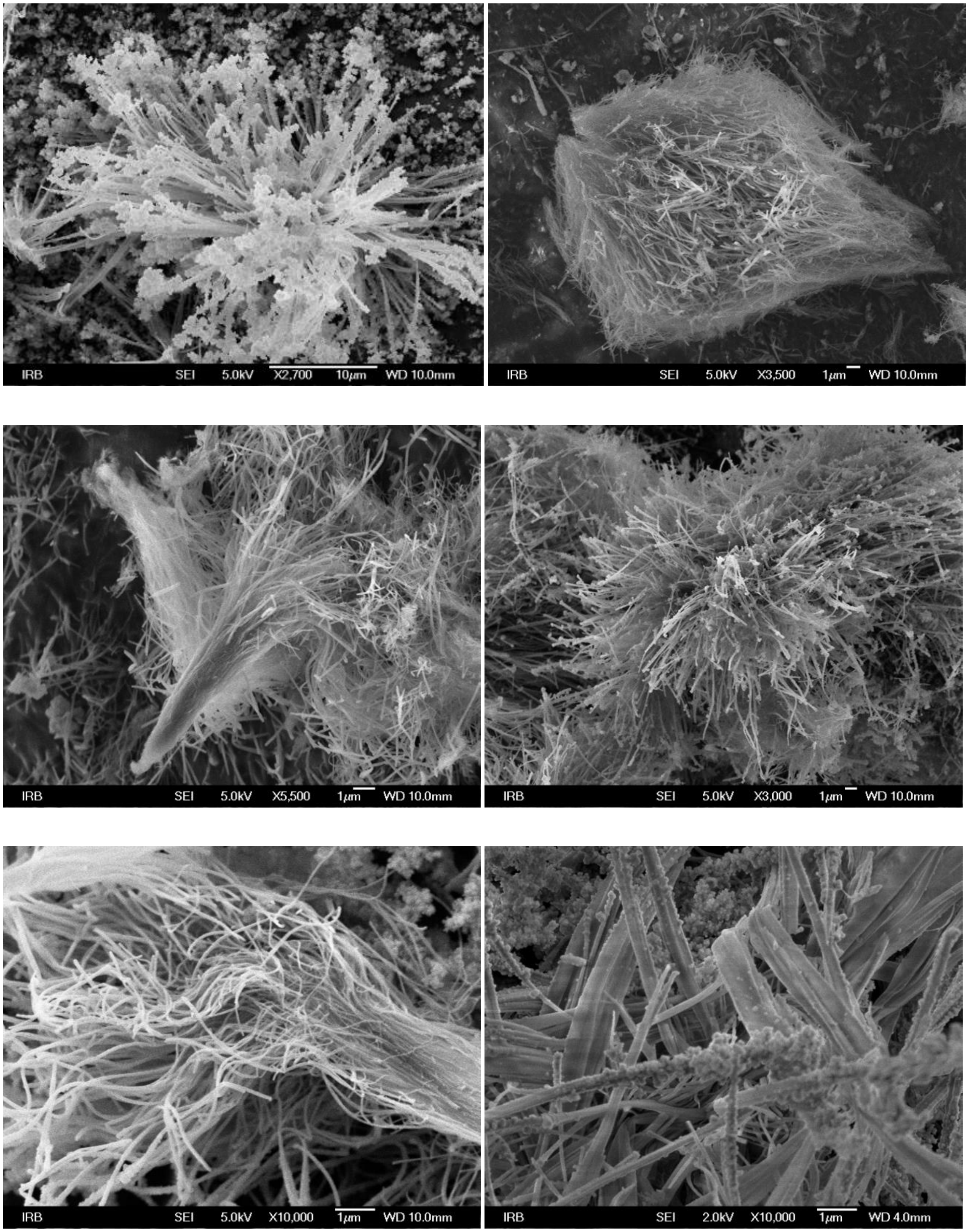
IRB SEI 5.0kV X2,700 10μm WD 10.0mm
IRB SEI 5.0kV X3,500 1μm WD 10.0mm
IRB SEI 5.0kV X5,500 1μm WD 10.0mm
IRB SEI 5.0kV X3,000 1μm WD 10.0mm
IRB SEI 5.0kV X10,000 1μm WD 10.0mm
IRB SEI 2.0kV X10,000 1μm WD 4.0mm

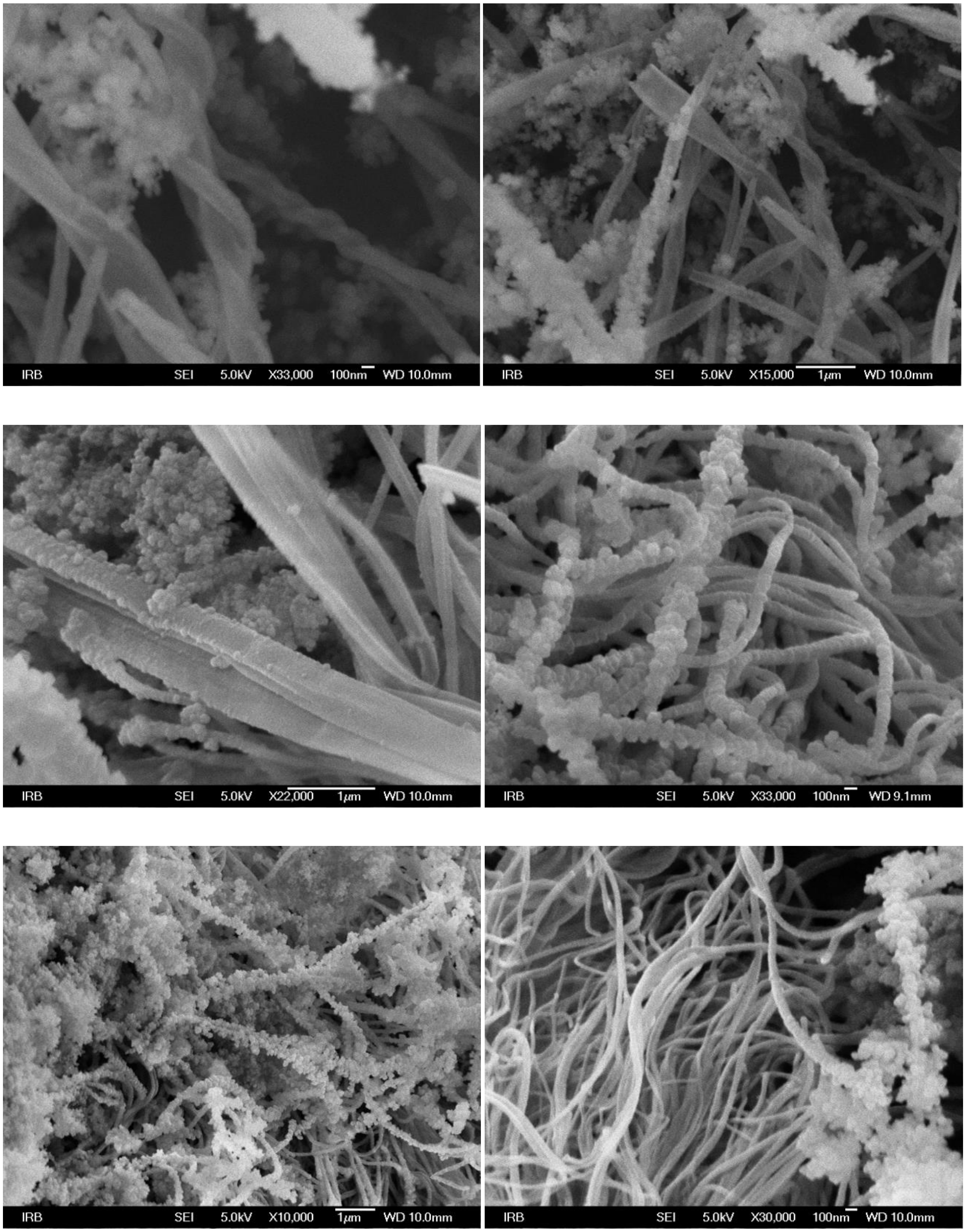
IRB SEI 5.0kV X33,000 100nm WD 10.0mm
IRB SEI 5.0kV X15,000 1μm WD 10.0mm
IRB SEI 5.0kV X22,000 1μm WD 10.0mm
IRB SEI 5.0kV X33,000 100nm WD 9.1mm
IRB SEI 5.0kV X10,000 1μm WD 10.0mm
IRB SEI 5.0kV X30,000 100nm WD 10.0mm

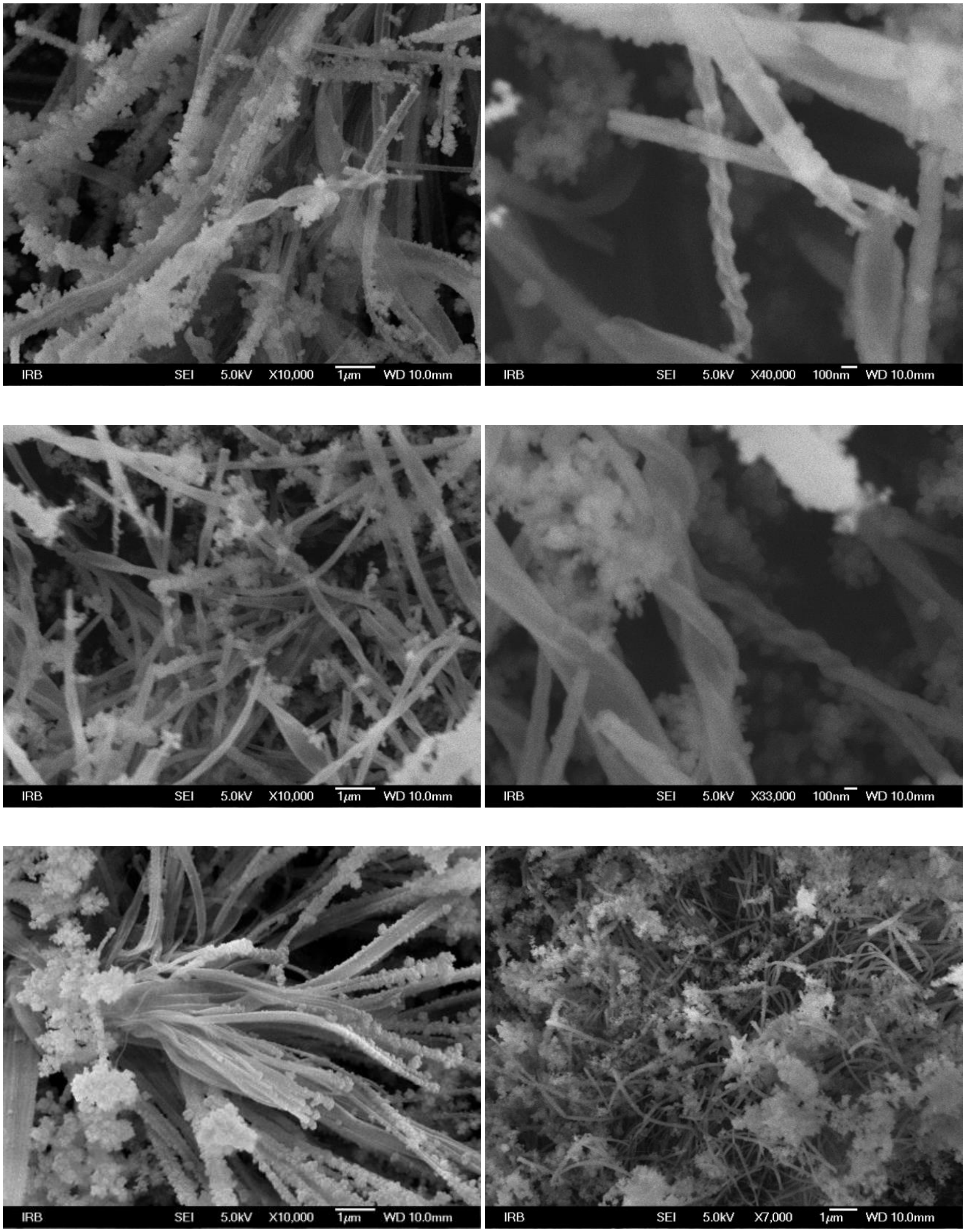

Figure S2. SEM micrography form obtained by gamma ray polymerization of **1a**/DMF-H_2O gel network

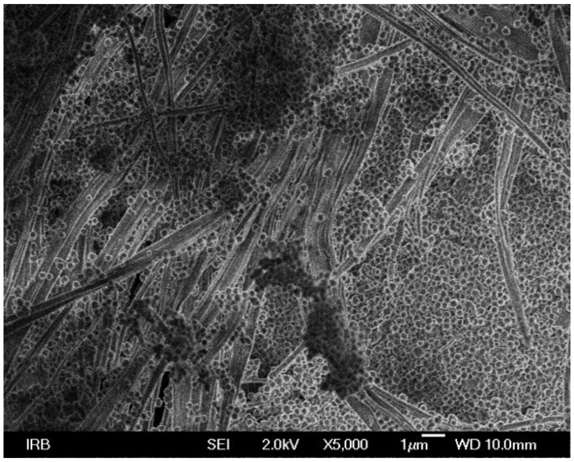
IRB SEI 2.0kV X5,000 1μm WD 10.0mm
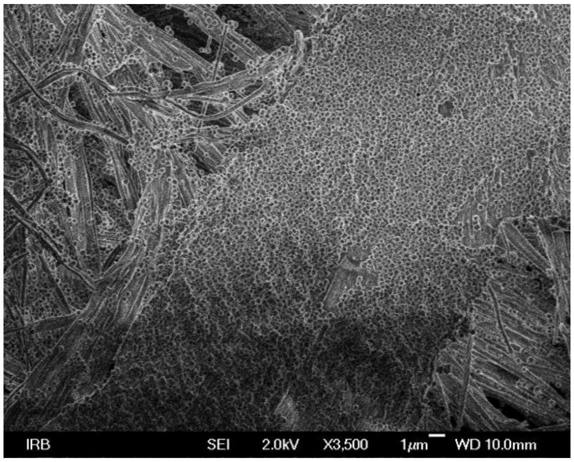
IRB SEI 2.0kV X3,500 1μm WD 10.0mm
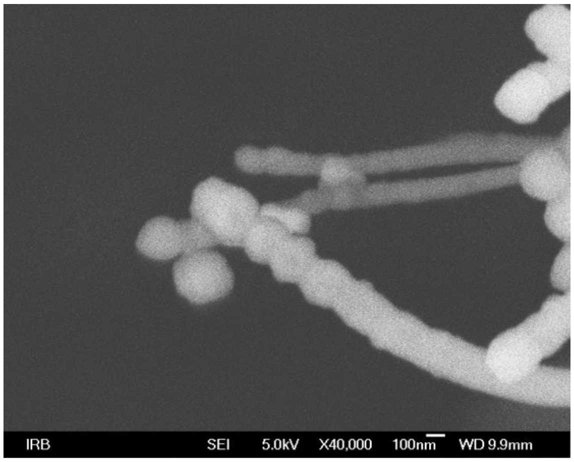
IRB SEI 5.0kV X40,000 100nm WD 9.9mm
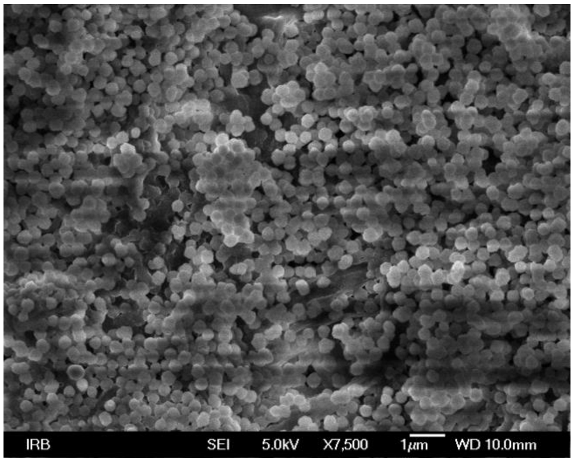
IRB SEI 5.0kV X7,500 1μm WD 10.0mm
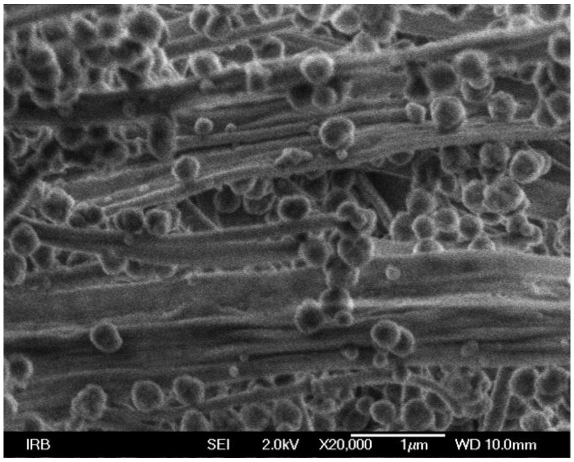
IRB SEI 2.0kV X20,000 1μm WD 10.0mm
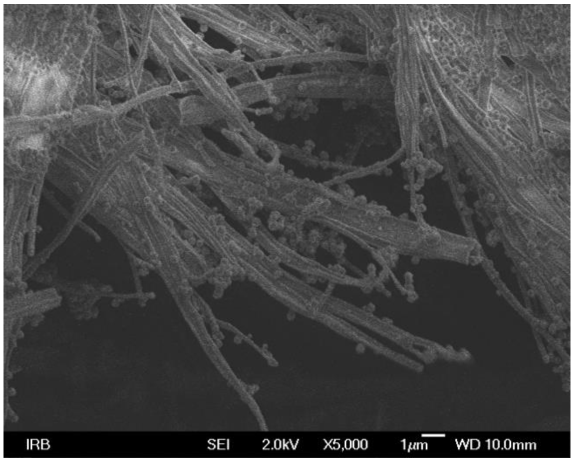
IRB SEI 2.0kV X5,000 1μm WD 10.0mm

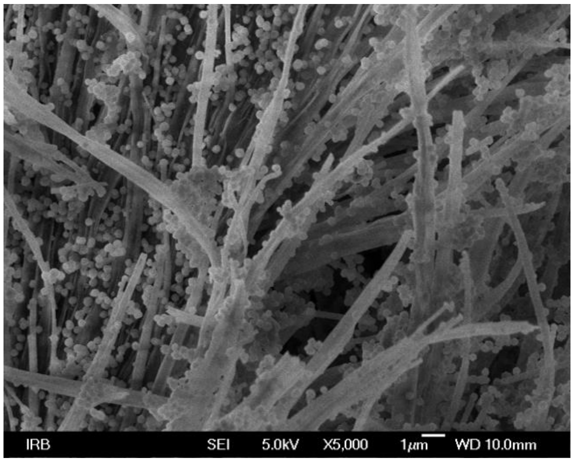

Figure S3. SEM micrography of gamma ray polymerization of **1a**/DMF-H_2O gel network on a glass slice

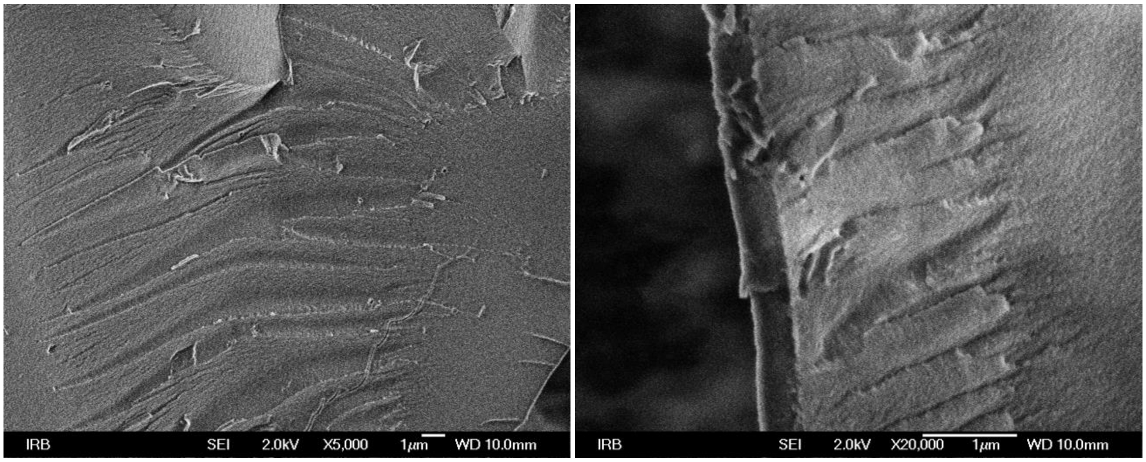

Figure S4. SEM micrography of product from the gamma ray polymerization **1a**/toluene gel network

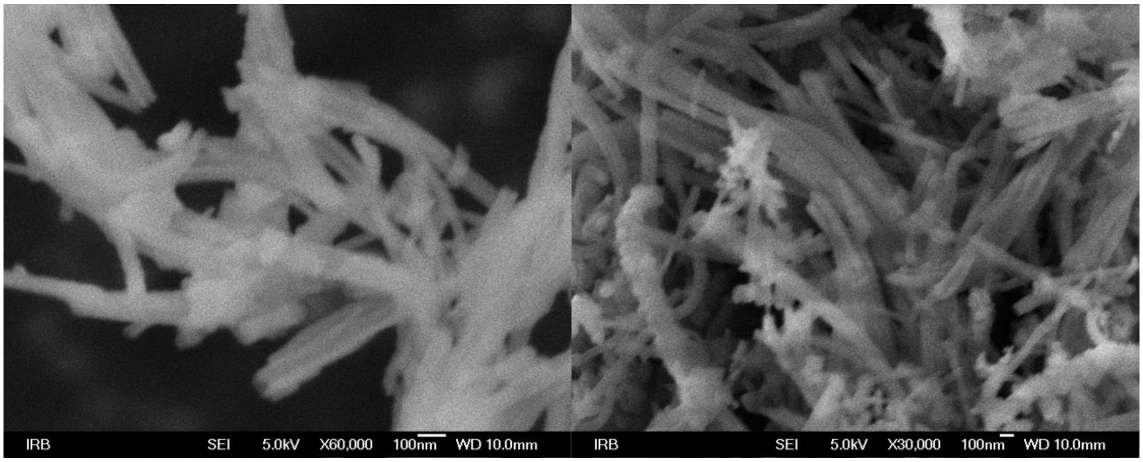

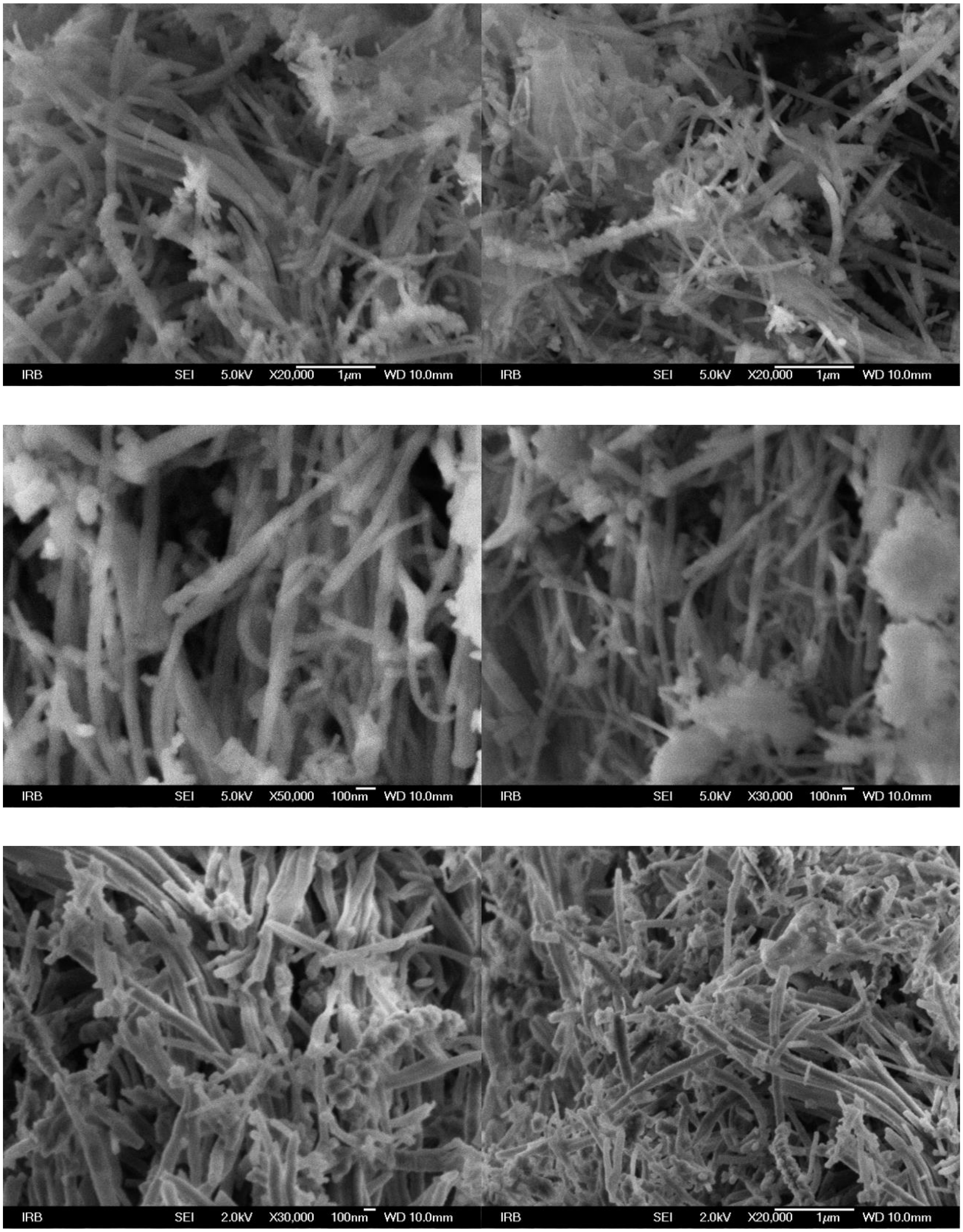

Figure S5; SEM micrography of UV polymerization from **1a**/DMF-H_2O gel network.

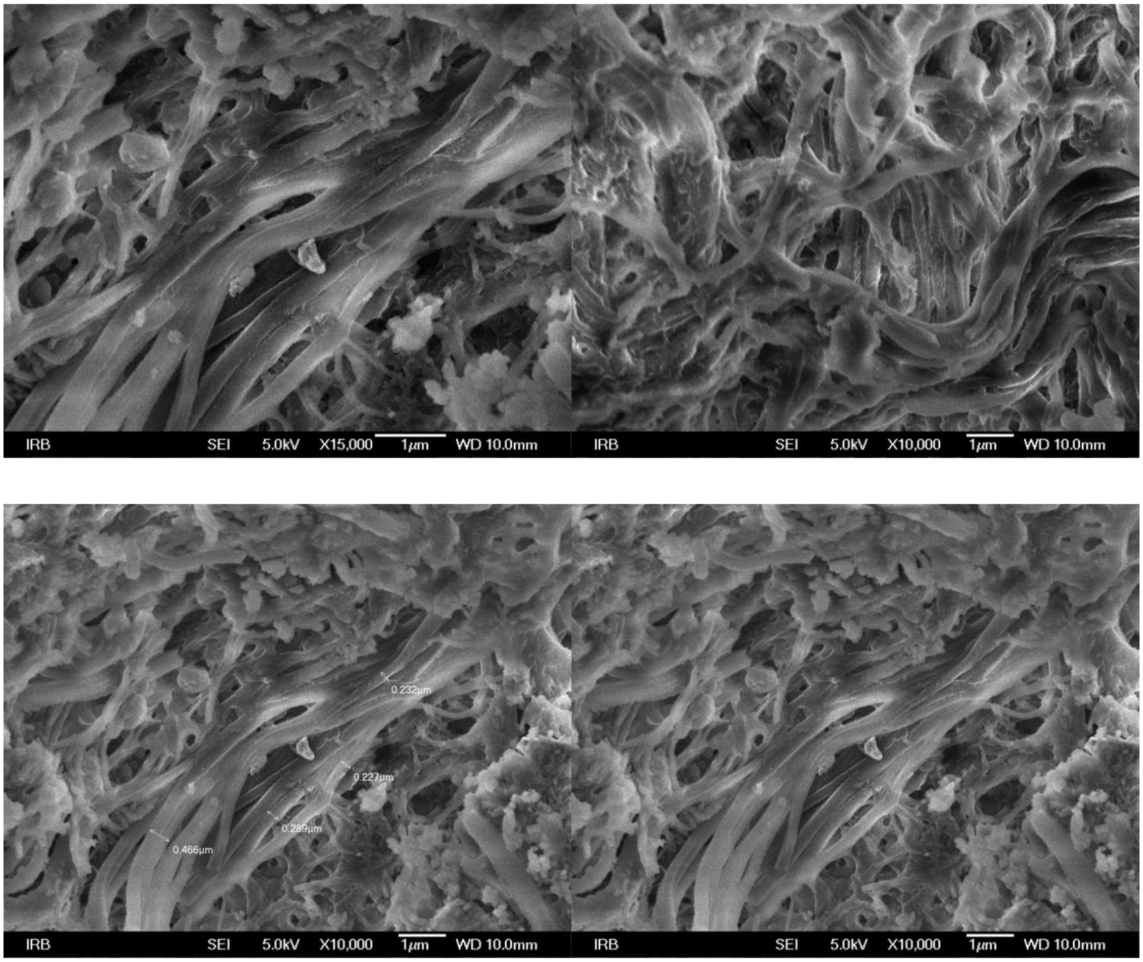

Figure S6; SEM micrography of UV polymerization from **2a**/ toluen gel network

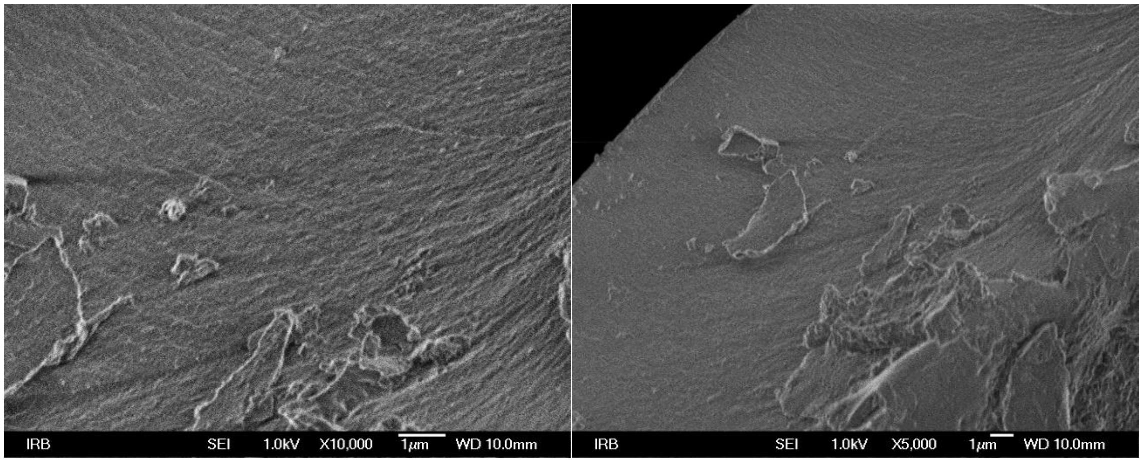

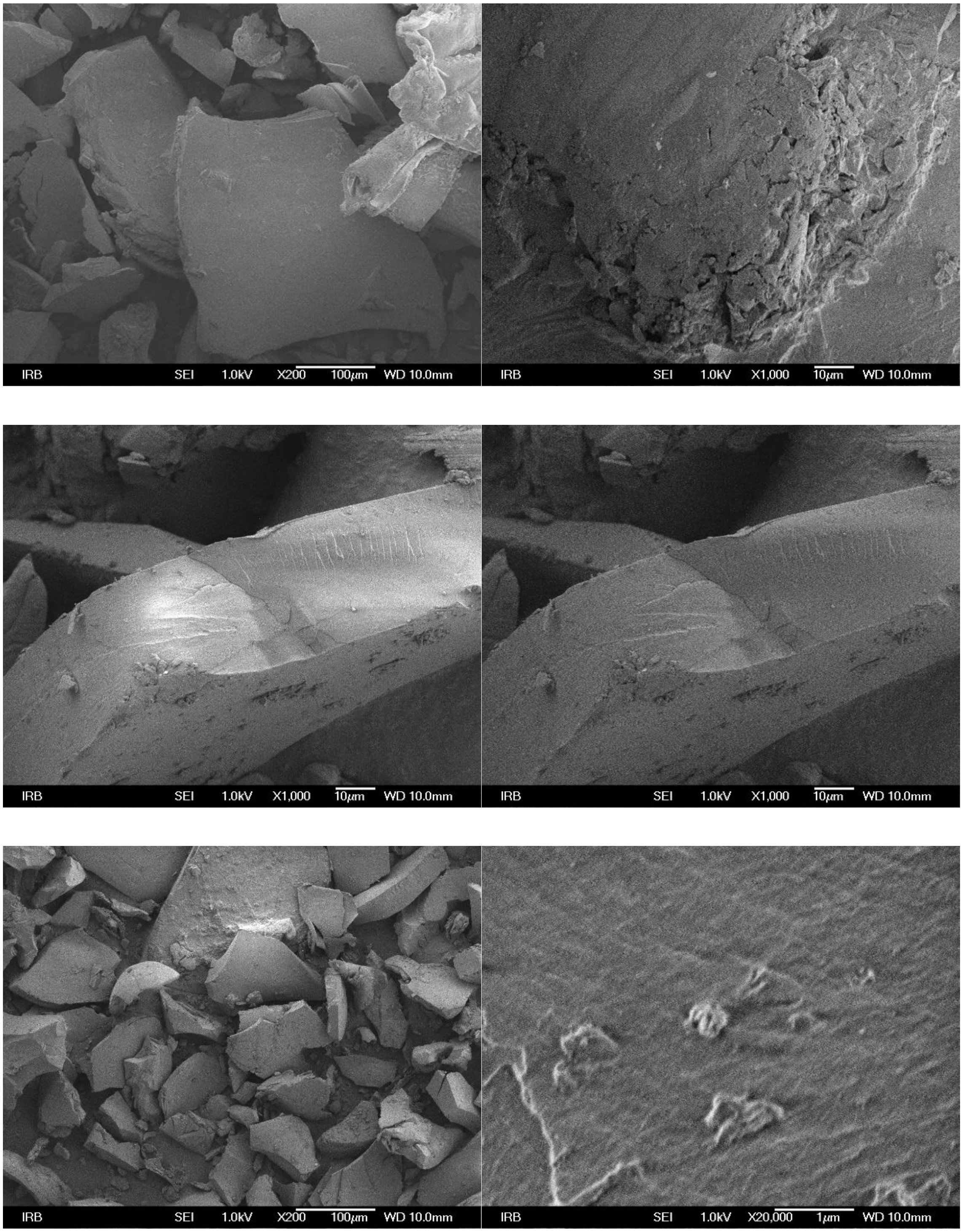

Figure S7. SEM micrography of UV polymerization CH_2Cl_2 solution of compound **1a**

4. FTIR Measurements

Table S2; *Characteristic FTIR bands (cm^{-1}) of **1a**, **2a** (gel, crystal, product gamma, irradiation in solution).*

	NH	*CO(OR)*	*amide I*	*amide II*
**1a**/toluene gel	3385<br>3288	1760	1647<br>1638	solvent
**1a**/crystal	3317	1772<br>1753<br>1742	1645<br>1631	1541
**1a**/$CDCl_3$ solution	3425<br>3298	1751	1666<br>1647	ca 1513
**1a**/toluene polymerisation	3367<br>3292	1747	1647	1535
**2a**/toluene gel	3291<br>3266	1749	1635	-
**2a**/crystal	3292<br>3268	1750	1639	1554
**2a**/$CDCl_3$ solution	34213, 311br		1750	1655<br>1647

a)

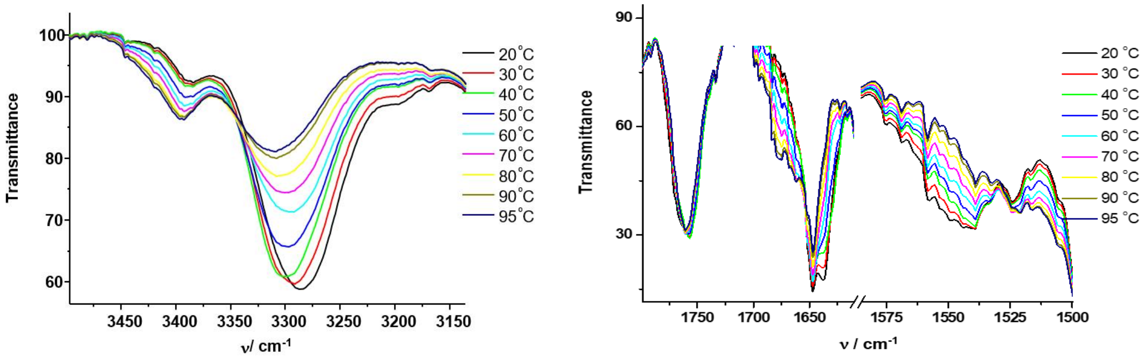

b)

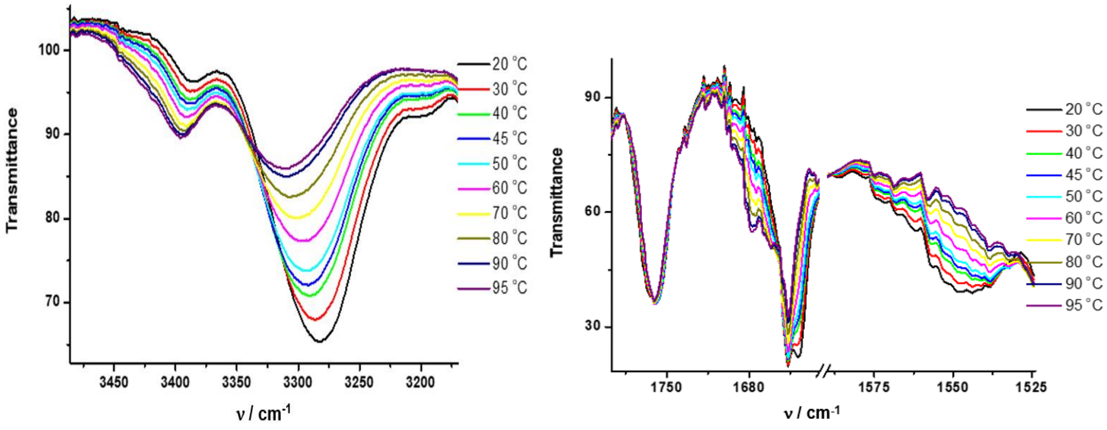

c)

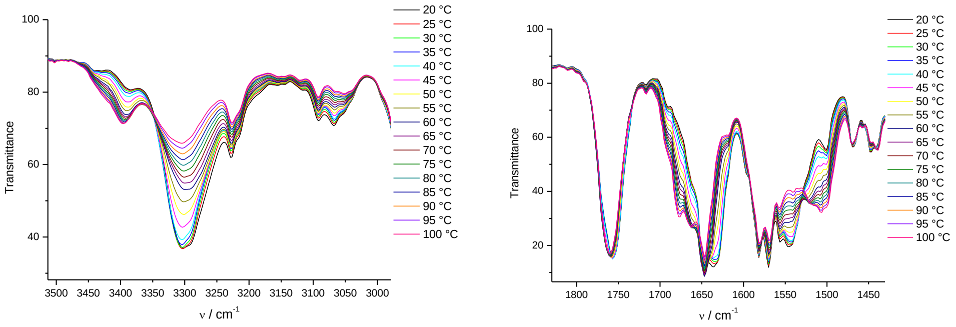

Figure S8. Temperature dependent FTIR spectra of a) **1a**/toluene gel (ultrasound induced gelation) b) **1a**/toluene-d_8 gels (without application of ultrasound) in the temperature range of 20-95 ºC and c) **1a**/toluene gel with 5 % mol benzophenone in the temperature range of 20-100 ºC

5. 2D NOESY spectra

a)

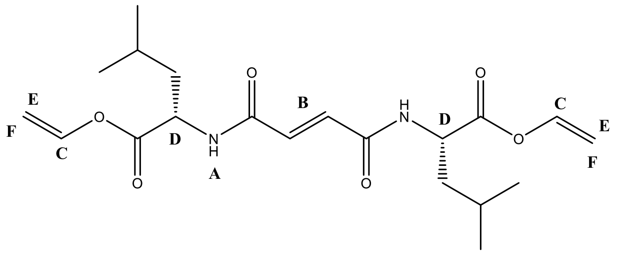

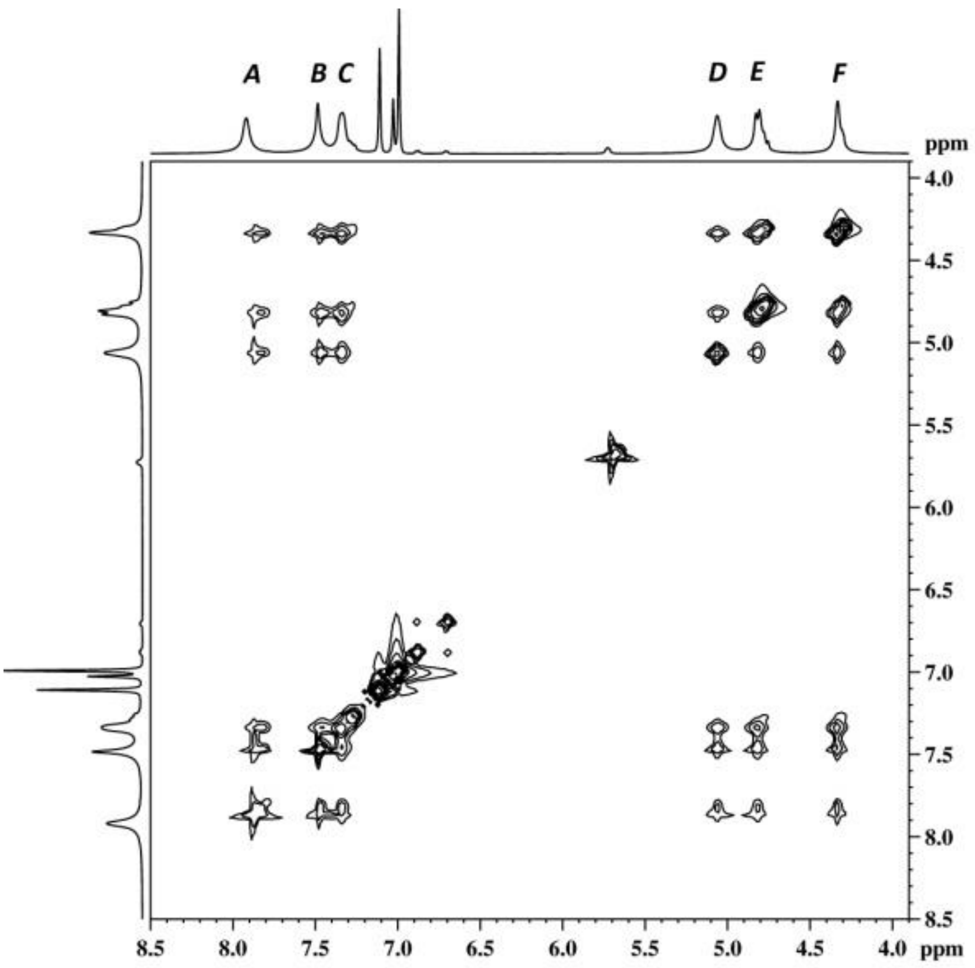
A
B C
D E
F
ppm
4.0
4.5
5.0
5.5
6.0
6.5
7.0
7.5
8.0
8.5
8.5 8.0 7.5 7.0 6.5 6.0 5.5 5.0 4.5 4.0 ppm

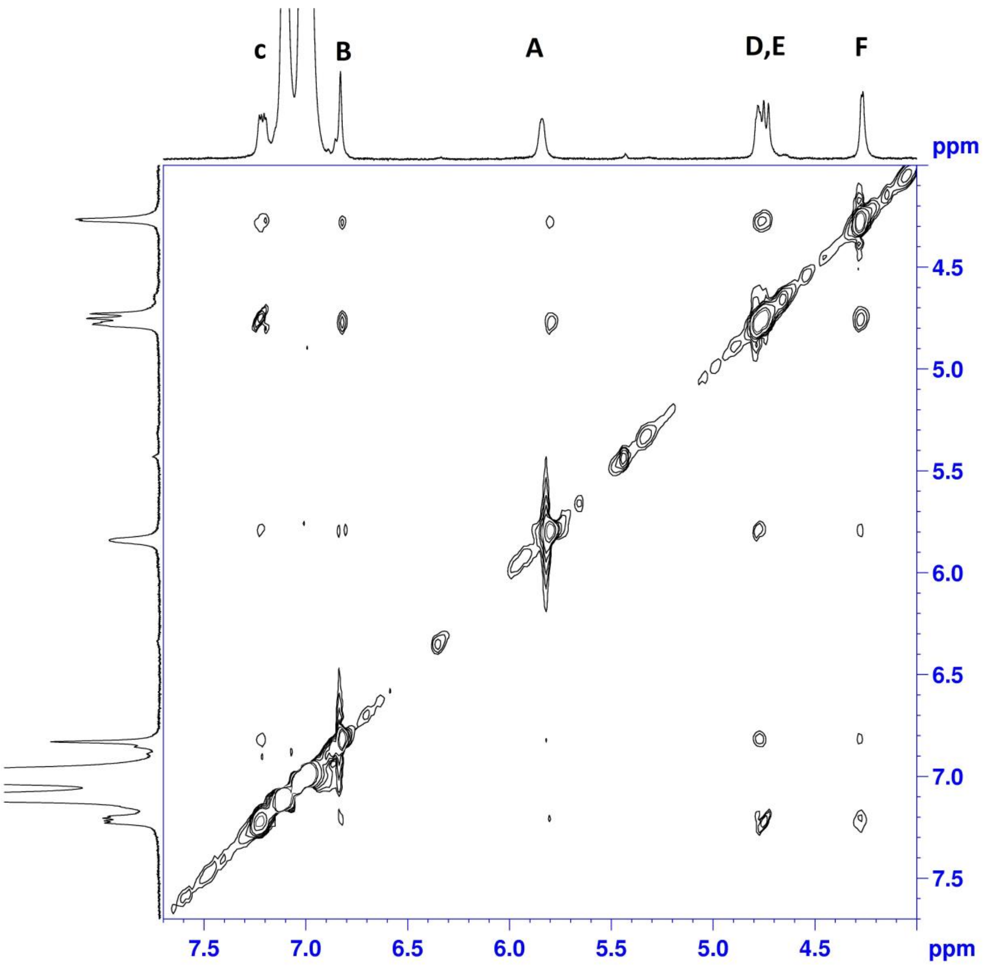
c
B
A
D,E
F
ppm
4.5
5.0
5.5
6.0
6.5
7.0
7.5
7.5
7.0
6.5
6.0
5.5
5.0
4.5
ppm

b)

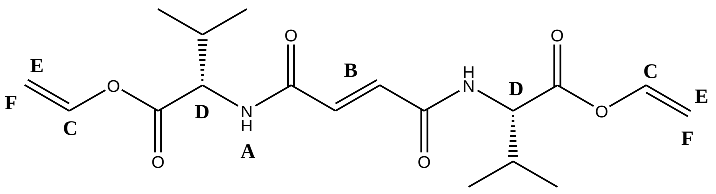
E
F
C
O
O
D
N
H
A
O
B
O
H
N
D
O
O
C
E
F

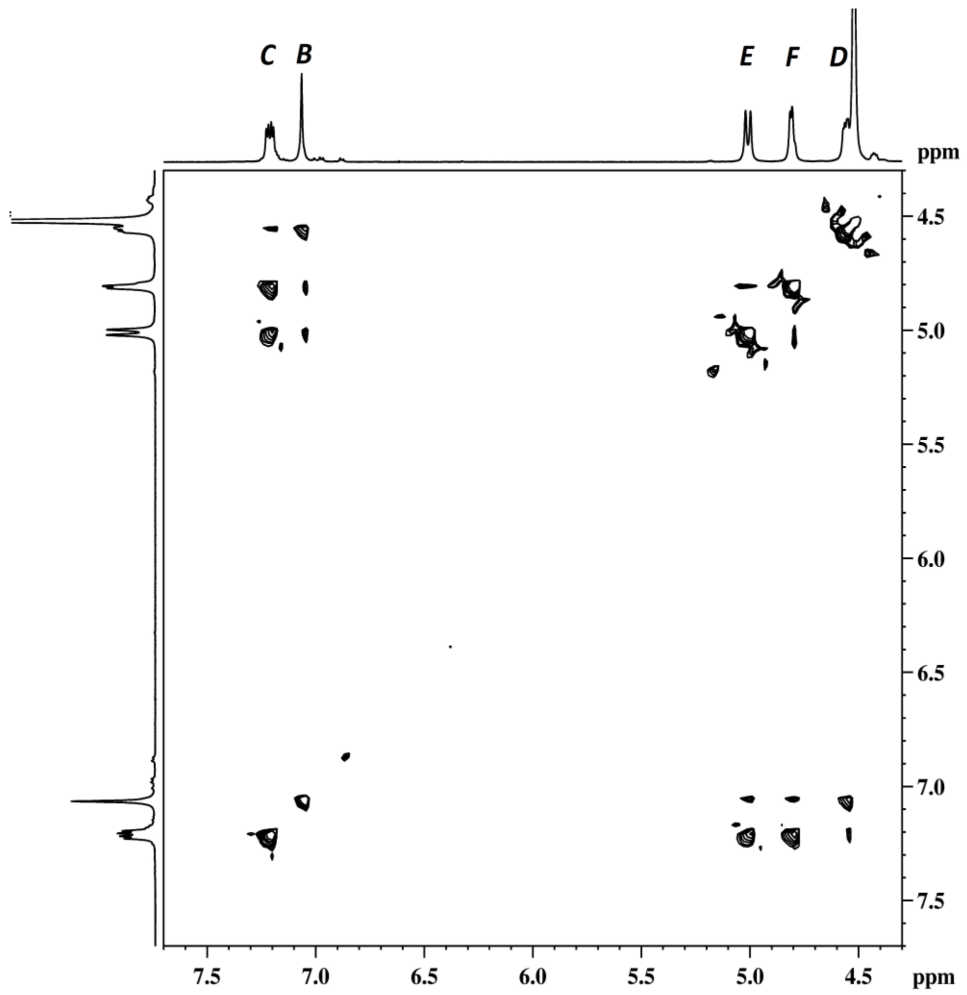
C
B
E
F
D
ppm
4.5
5.0
5.5
6.0
6.5
7.0
7.5
7.5
7.0
6.5
6.0
5.5
5.0
4.5
ppm

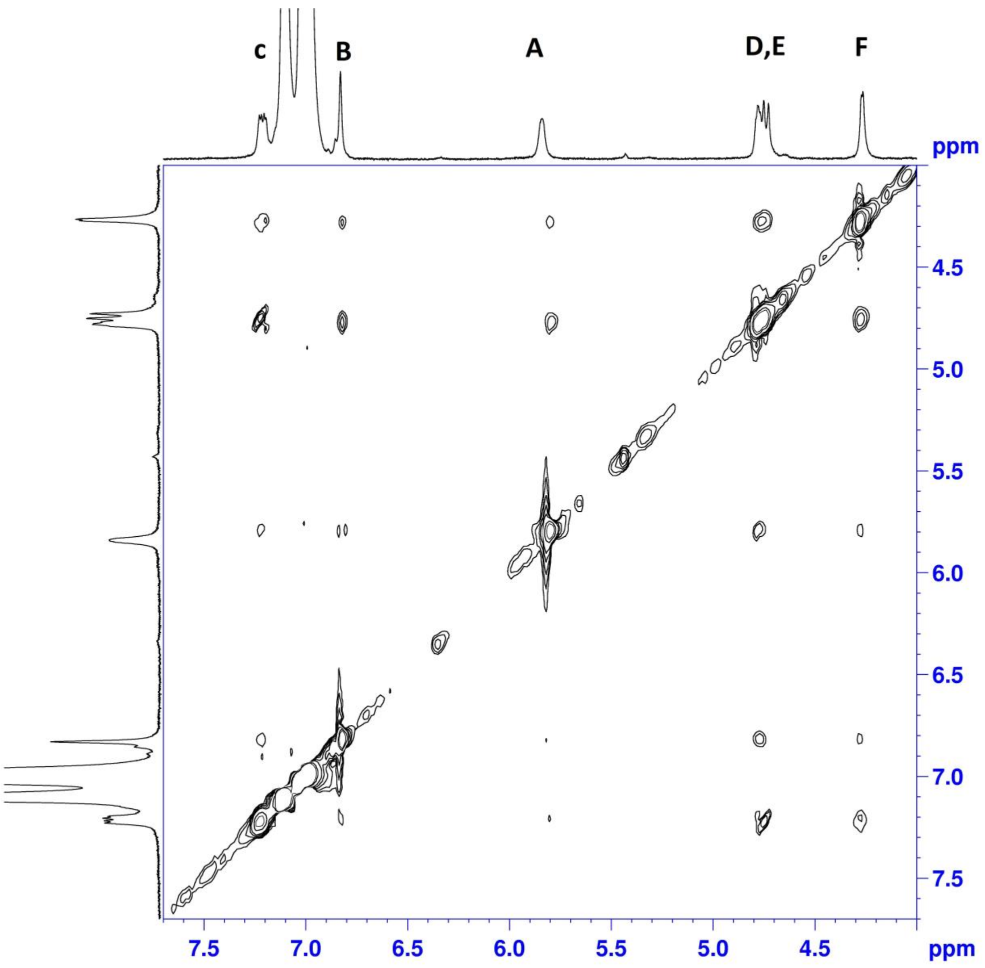
c
B
A
D,E
F
ppm
4.5
5.0
5.5
6.0
6.5
7.0
7.5
7.5
7.0
6.5
6.0
5.5
5.0
4.5
ppm

c)

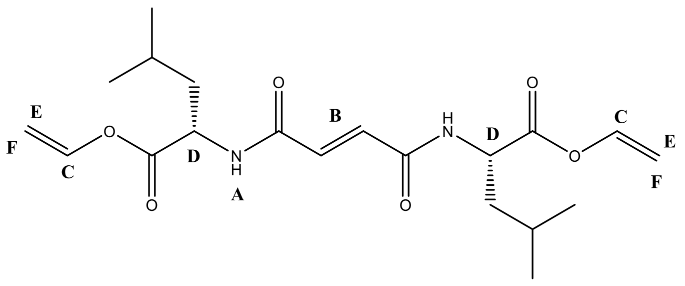
E
F
C
O
O
D
N
H
A
O
B
O
H
N
D
O
O
C
E
F

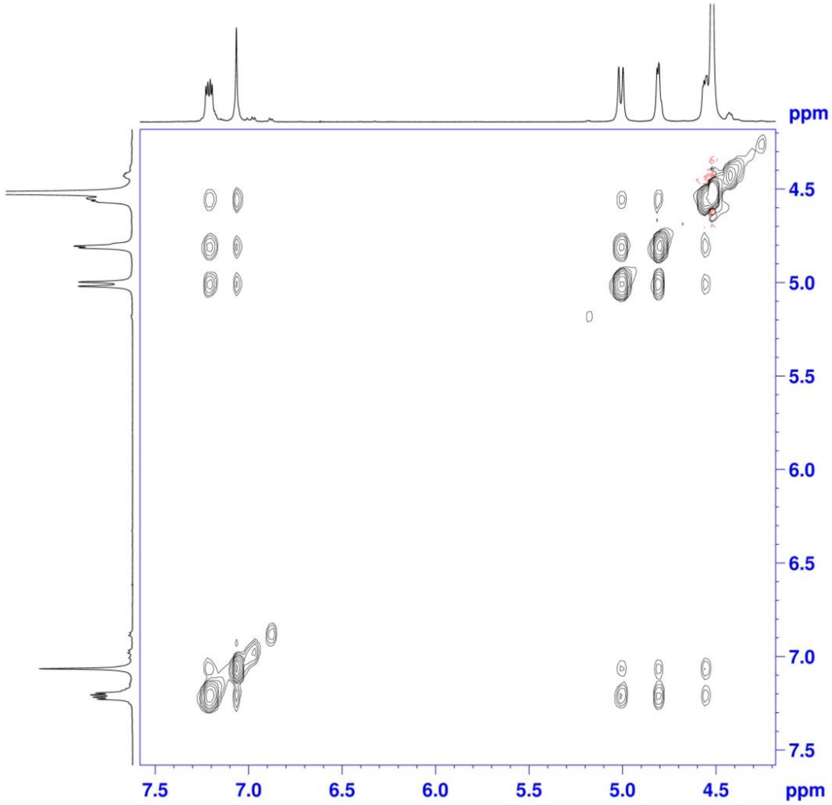

Figure S9. NMR; Selected region of 2D NOESY spectra of a) **1a**/toluene gel; b) **2a**/toluene gel; c) **1a**/DMF-H_2O gel; [***A***=NH, ***B*** **CH**=**CH**$_{(fum)}$, ***C*** O-**CH**=$CH_{2,\ viny}$, ***D*** CH*, ***E*** and ***F*** O-CH=**CH_2**$_{,\ vinyl}$ protons.

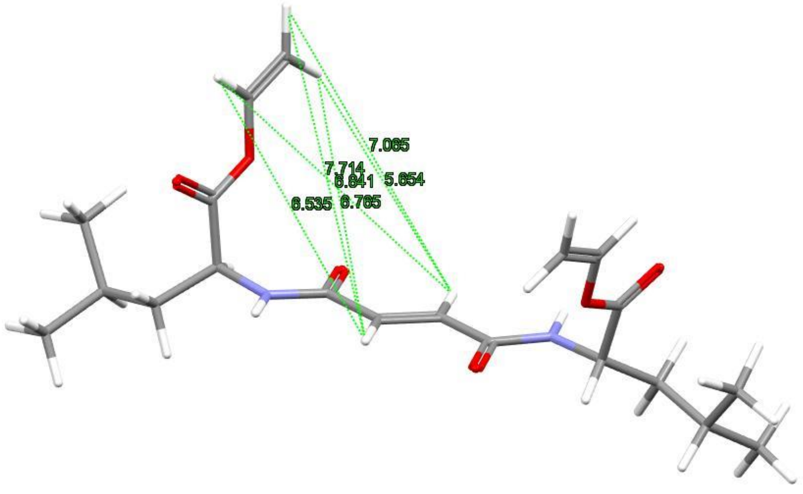

Figure S10. Model of **1a**. Distances H – H (vinyl ester : vinyl fum) in fully minimized the lowest energy conformations of **1a** (**C – B:** 6.6 – 6.8 Å; **E – B:** 5.6 – 6.7 Å;**F – B** 7.0 - 7.7 Å)

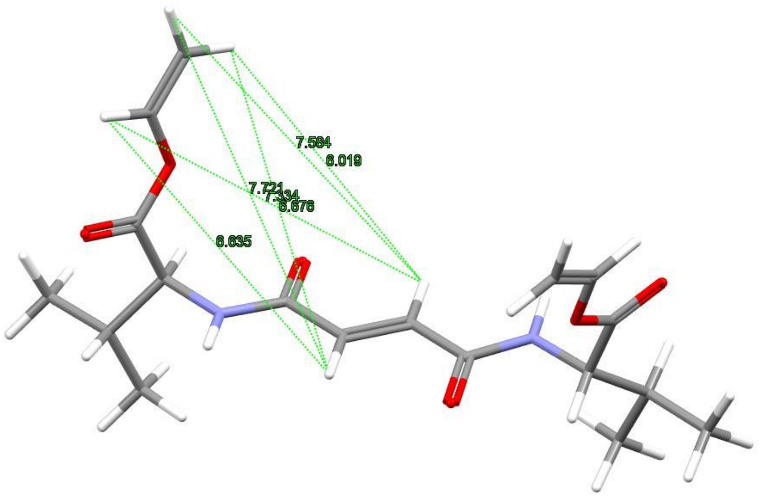

Figure S11. Model of **2a**. Distances H – H (vinyl ester : vinyl fum) in fully minimized the lowest energy conformations of **2a** (**C – B** 6.6 – 7.3 Å; **E – B** 6.0 – 6.3 Å; **F – B** 7.2 - 7.5 Å)

5. Molecular modelling

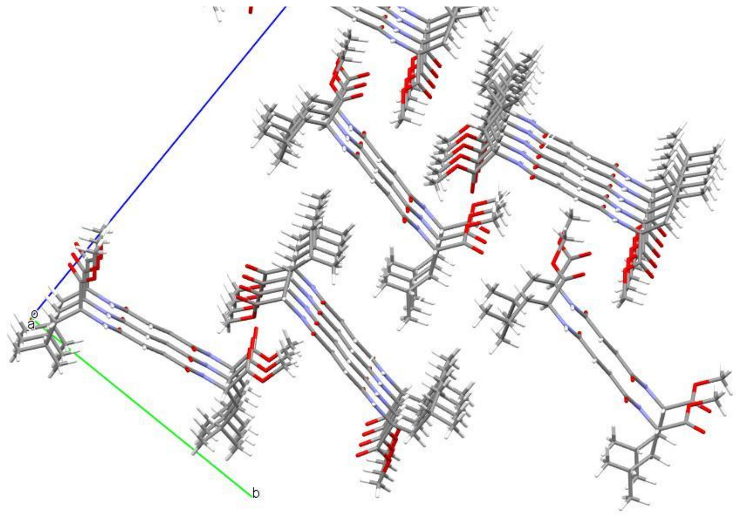

Figure S12. Crystal structure of *N,N'*-bis[(2*S*)-1-methoxy-3-methyl-1-oxobutane-2-yl] fumaramide (**1b**); a bilayer of molecules linked by H-bonds (1D Fum NH – O=C); 3D all *i*-Bu groups (L-Leu) are oriented are oriented towards each other, OMe groups are located on the other side of the bilayer[69].

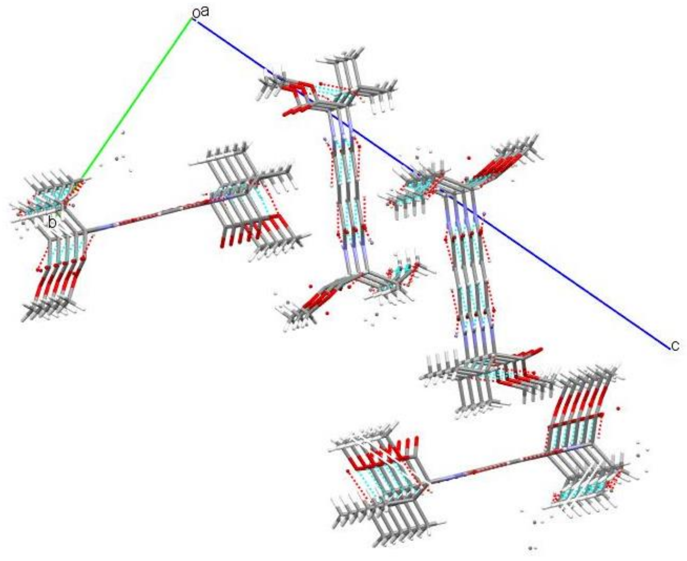

Figure S13. Crystal structure *N,N'*-bis[(2S)-1-methoxy-4-methyl-1-oxopentane-2-yl] fumaramide (**2b**) (1D H-bonds Fum NH – O=C); the layers are vertically oriented like a herringbone pattern, 3D *i*-Pr group (L-Val) together with OMe oriented in a “cavity”, OMe groups oriented towards to another OMe (CCDC: 2124266.).

a)

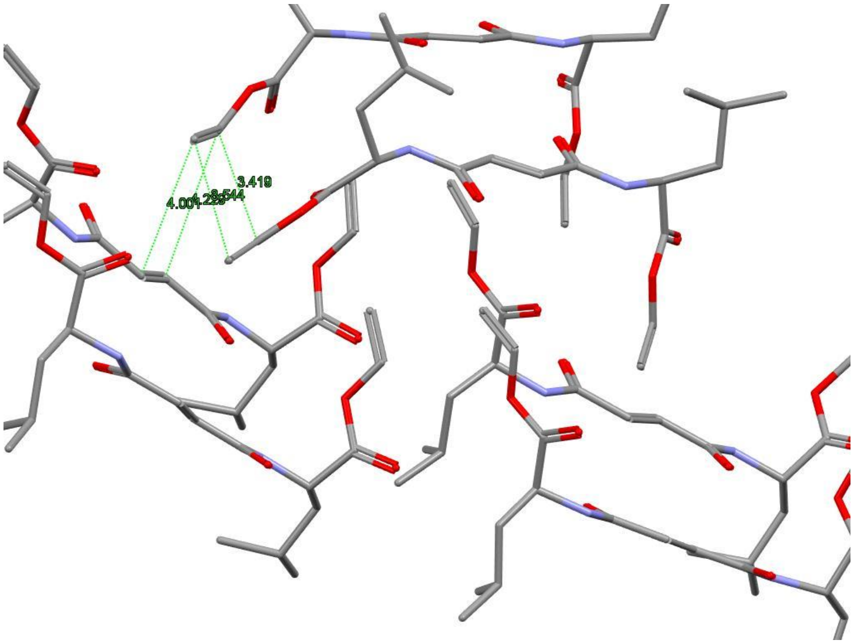

b)

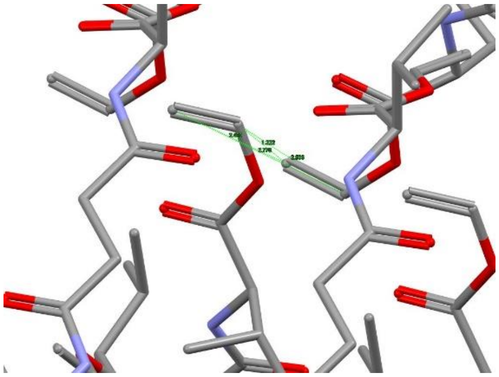

Figure S14. a) Distances reactive groups (C=C) under 4 Å in the model of favourable packing of the 6 molecules linked by amide H-bonds (NH-O = C) (**1a**) obtained by molecular modelling. Hydrogen atoms are omitted for clarity. b) enlarged part of the structure.

a)

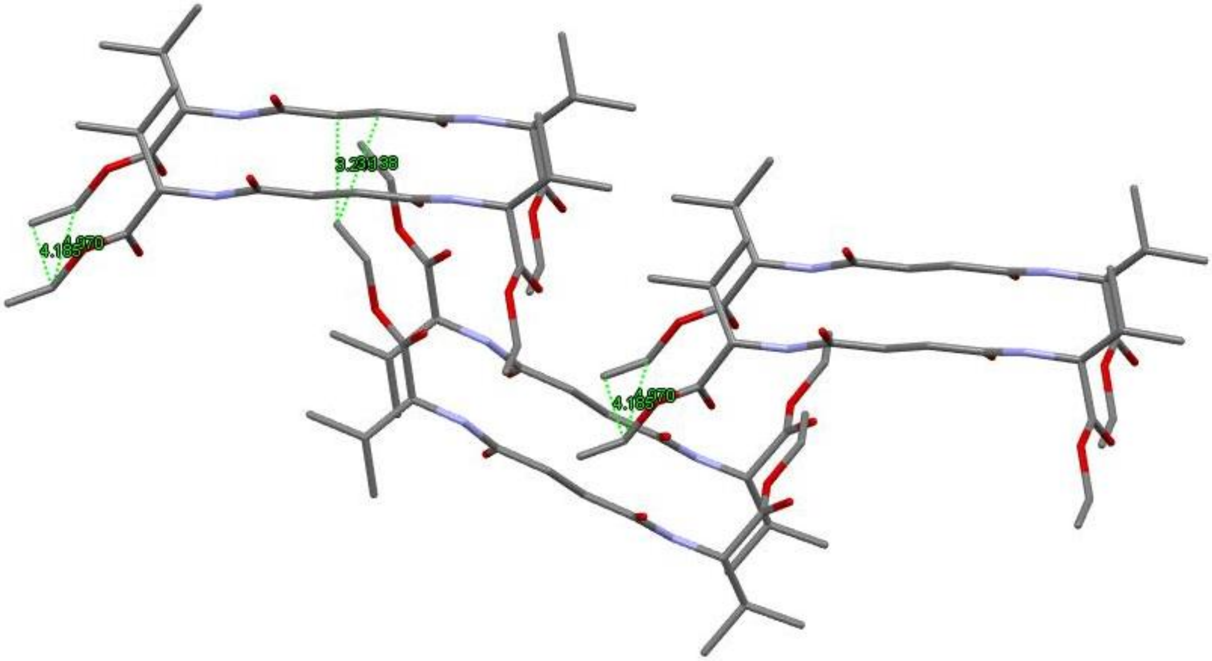

b)

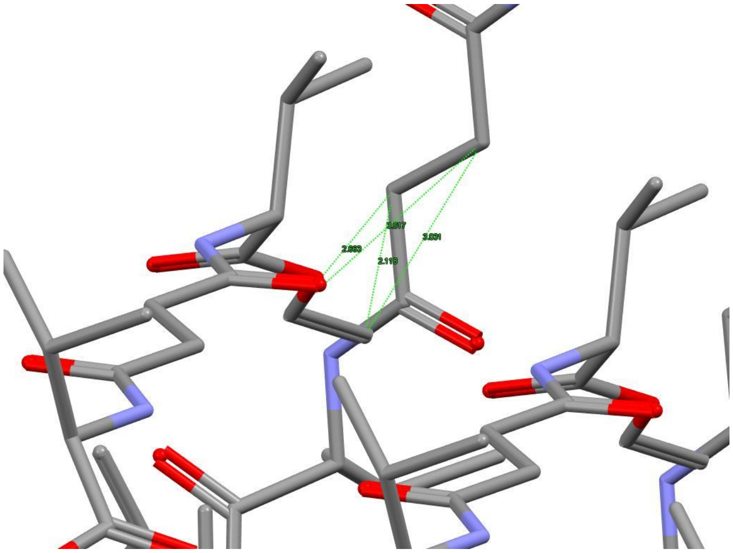

Figure S15. Distances reactive groups (C=C) ~ 4 Å in the model of favourable packing of the 6 molecules linked by amide H-bonds (NH-O = C) (**2a**) obtained by molecular modelling. Hydrogen atoms are omitted for clarity. b) enlarged part of the structure.

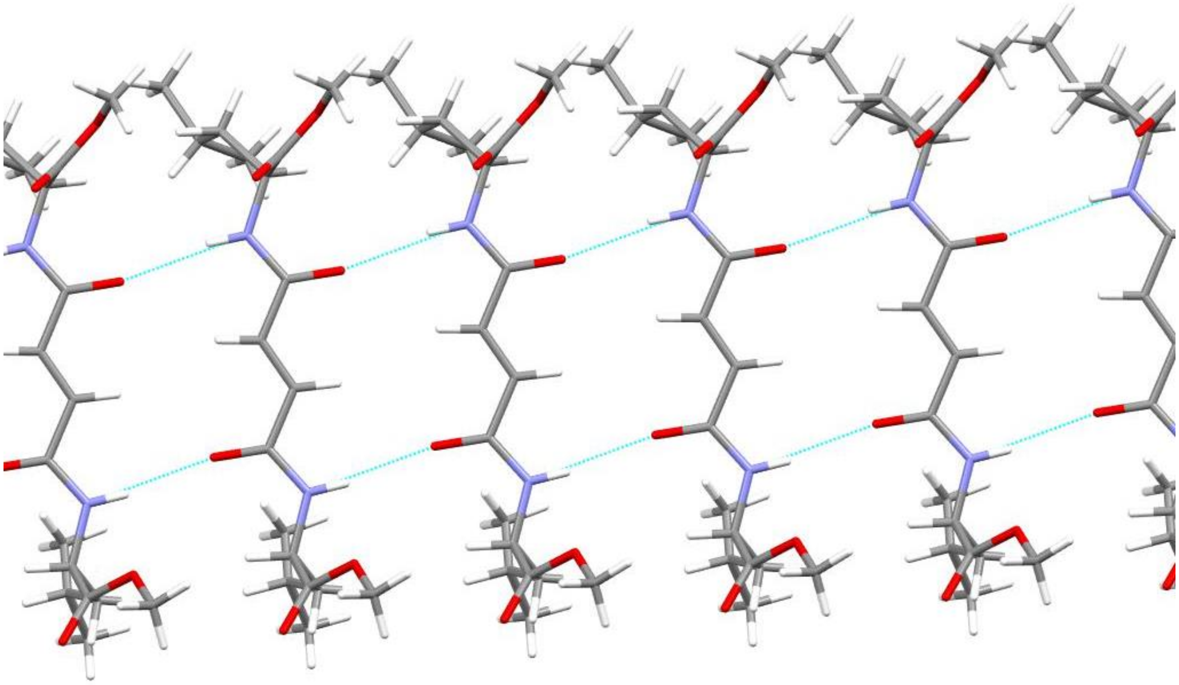

Figure S16. Hydrogen bond pattern in crystal structure of *N,N′*-bis[(2*S*)-1-methoxy-3-methyl-1-oxobutane-2-yl] fumaramide (**1b**).[69]

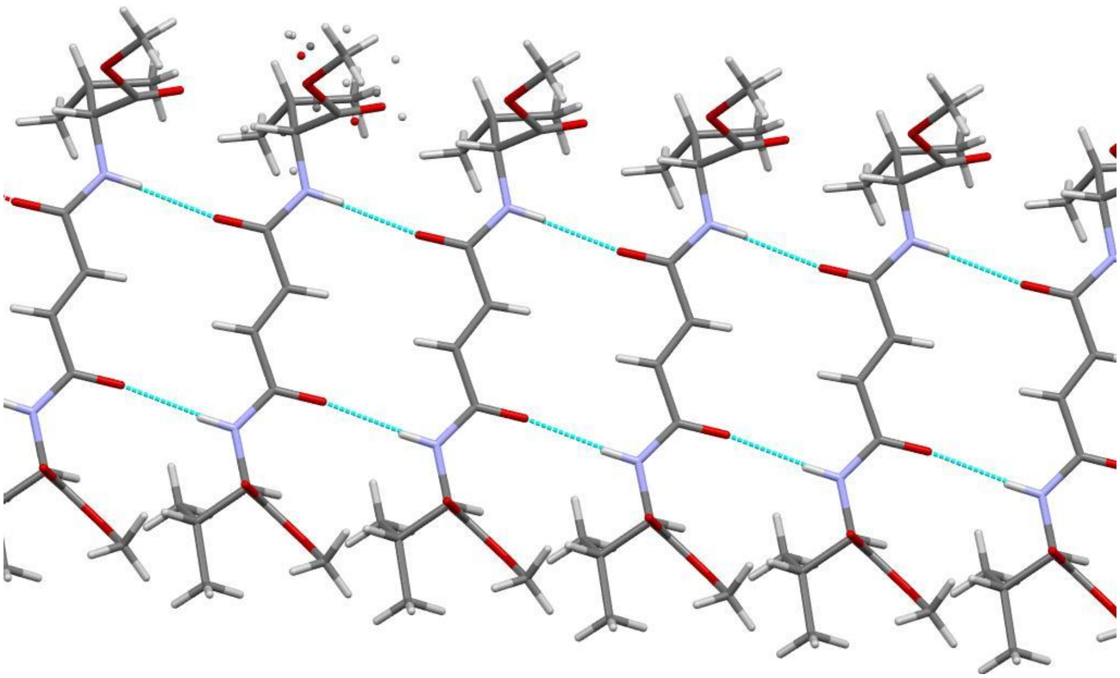

Figure S17. Hydrogen bond pattern in crystal structure of *N,N′*-bis[(2S)-1-methoxy-4-methyl-1-oxopentane-2-yl] fumaramide (**2b**), (CCDC: 2124266).

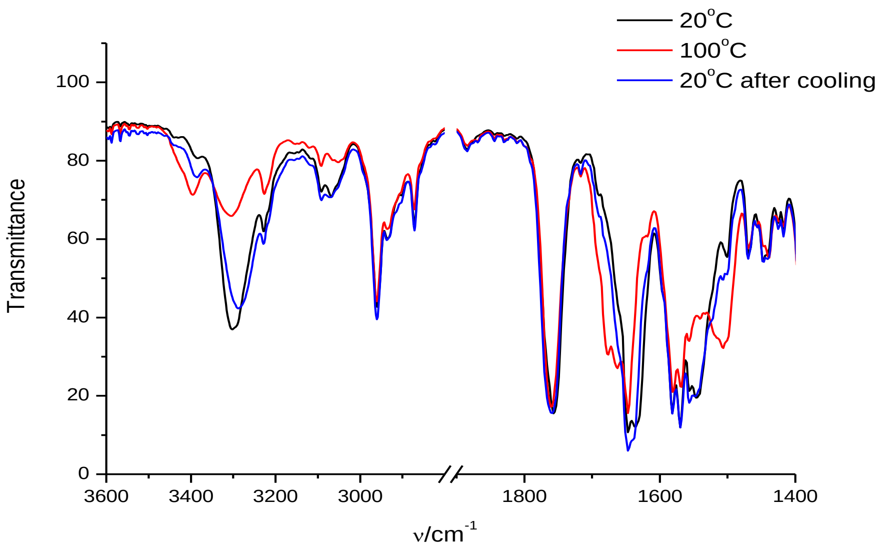

Figure S18 Temperature FTIR spectra **1a**/toluene-d_8 (c= 0.23 M) before heating (black) at 100 °C (red) and after cooling (blue)

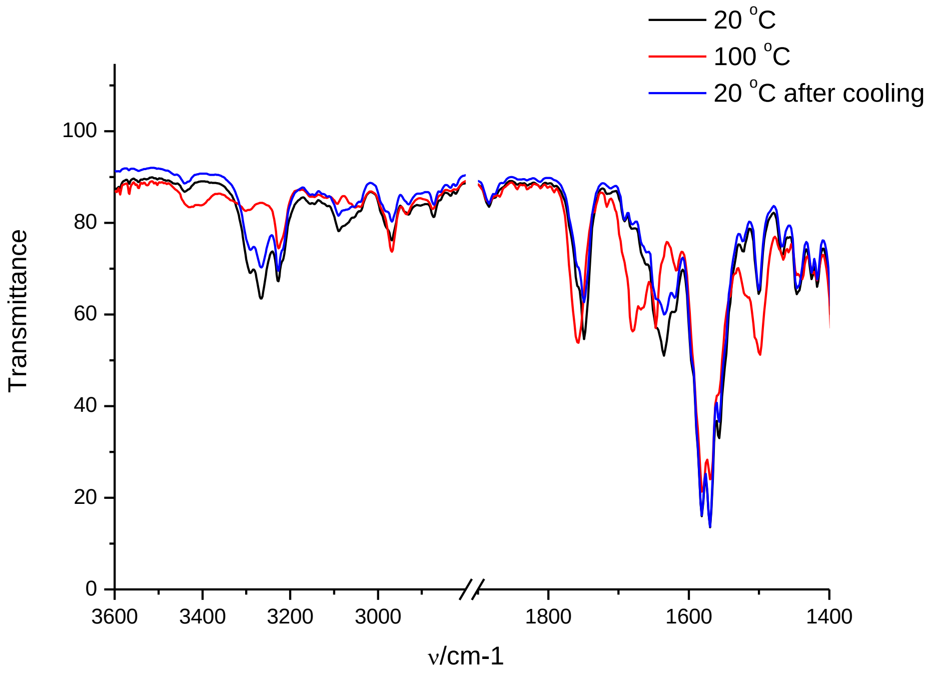

Figure S19 Temperature FTIR spectra **2a**/toluene-d_8 (c=4.2x10^{-2} M) gels) before heating (black) at 100 °C (red) and after cooling (blue)

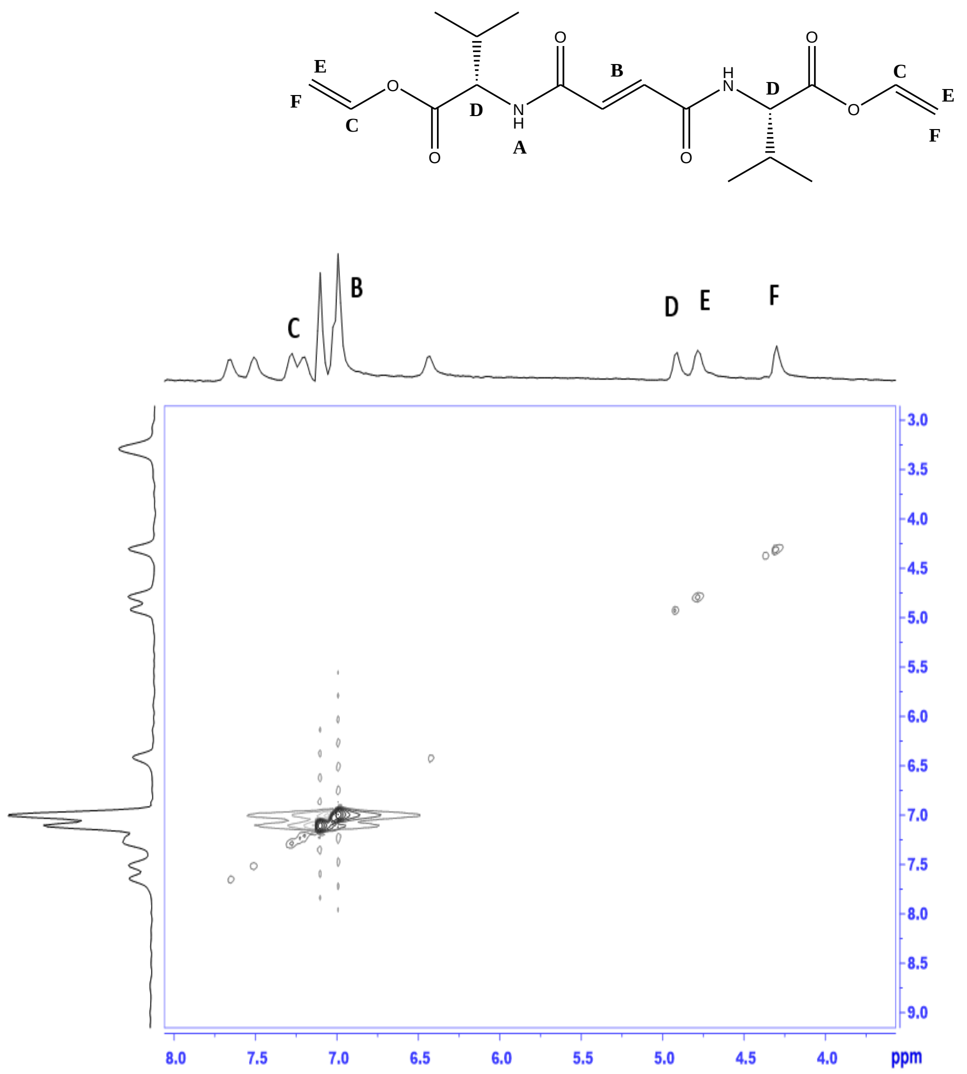

Figure S20. Selected region of 2D NOESY spectra of **2a**/toluene gel at 40 °C; [***A***=NH, ***B*** **CH**=**CH**(fum), ***C*** O-**CH**=CH_{2}, viny, ***D*** CH*, ***E*** and ***F*** O-CH=**CH_2**, vinyl protons
